# Couples and parenting dynamics during Covid-19 pandemic: A systematic review of the literature

**DOI:** 10.1371/journal.pone.0315417

**Published:** 2025-02-18

**Authors:** Maria Leonor Sentieiro, Luana Cunha Ferreira, Mariana Pires de Miranda, Beatriz Araújo Vitória

**Affiliations:** 1 Faculty of Psychology, University of Lisbon, Lisbon, Portugal; 2 Faculty of Psychology, University of Coimbra, Coimbra, Portugal; 3 CICPSI, Faculdade de Psicologia, Universidade de Lisboa, Alameda da Universidade, Lisboa, Portugal; 4 Ispa–Instituto Universitário, Lisbon, Portugal; University of Nicosia, CYPRUS

## Abstract

The COVID-19 pandemic constituted a public health crisis bound to impact couples, parents, and families globally. However, the literature on the impacts of COVID-19 in families is not yet integrated. This systematic review aims to (1) provide an overview of how the COVID-19 pandemic potentially changed family systems, especially the couples and parenting dynamics, as well as (2) integrate inconsistent findings, and, finally, (3) define new avenues for research and clinical practice. Inclusion and exclusion criteria were defined for this review. The data was collected in bibliographic databases using a combination of keywords. The study includes empirical research published in English, Spanish, Italian, or Portuguese, appearing in peer-reviewed journals, focusing on the impact of the COVID-19 pandemic on the functioning of different-gender or same-gender couples, married or cohabitating, or cohabitating parents with children aged 2 to 18, between April 2020 and December 2023. Within 381 articles, 58 studies met the inclusion criteria, which 50 were quantitative, four qualitative, and four mixed methods studies. A qualitative thematic analysis of the reviewed articles identified 12 categories that were organized by stress sources, mediators, and outcomes. There is consistent evidence across the literature showing some sources of stress during COVID-19, such as Gender Inequalities (e.g., Unequal Division of Household Duties and Lack of Support for Mothers), and External Stress. Also, we identified some themes on the mediators such as Coping Strategies (e.g., Dyadic Support, Communication as a Mediator and Adaptation to New Daily Routines) and finally as outcomes, Lower Psychological Well-Being, and Relational Growth. However, some inconsistencies were found in Relationship (In) Satisfaction, Sexual Functioning and Communication as an Outcome. Explorations of moderators showed that these inconsistencies should be understood in reference to (1) quality assessment (2) coping and (3) income. After conducting a comprehensive analysis of inconsistencies, our study revealed no notable differences in either relationship satisfaction or sexual frequency before and after the COVID-19 pandemic. Notably, a decline in sexual satisfaction was observed during the COVID-19 period. No conclusive associations were identified between income, relationship satisfaction, sexual satisfaction, sexual frequency, and stress related to COVID-19. Nonetheless, our observations indicated that individuals with moderate or high income, in conjunction with the implementation of effective coping strategies, exhibited a diminished impact of COVID-19-related stress on both relationship satisfaction and sexual functioning. Future studies should focus on a dyadic perspective at various stages of the family cycle, including longitudinal perspectives, as well as studies focused on the role of dyadic coping during times of crises. Findings must be considered with caution because not all studies had equal methodological rigor.

## Introduction

On the 18th of March 2020, the World Health Organization (WHO) declared COVID-19 a pandemic and a global public health emergency. Strict isolation measures were implemented worldwide to prevent the spread of the virus, resulting in lengthy lockdowns and social distancing measures impacting individuals, families, and society. Forced cohabitation led families to alter their dyadic and family-level dynamics. The objectives of this systematic review are to (a) describe relevant changes; (b) integrate inconsistent findings, and ultimately (c) define new avenues for both research and clinical practice.

### The family system and stress

The family system is a self-regulating system, that maintains stability through negative feedback and enables change through positive feedback [1]. A family consists of subsystems with specific roles and boundaries [2], like conjugality or parenting, and that adapts continually to maintain stability and allow change [2], underlying the importance of communication between family members [3].

The classic family life cycle starts with single adults forming couples, progressing through stages such as families without children, families with young children, and families with adolescents [4–6].

Is this literature review, we will particularly focus on three of the life cycles: families without children, families with young children and families with adolescents, as differences in household type during the lockdowns show that couples with children and lone parents were described as more materially vulnerable and subjectively constrained [7]. Across time, the family undergoes distinct cycles, steered by expected challenges and specificities that are inherent in its growth, in which families with adolescents require more flexibility in the family boundaries to accommodate their children’s independence while caring for the older generation [6].

However, there are also changes that families must cope with that go beyond the expected developmental process, such as coping with external stressors. Ecological Systems Theory [8] helps to understand the complex and multisystemic interplay between family system and subsystems, the societal and chronological contexts. This theory includes the microsystem (immediate environment), mesosystem (interactions between microsystems), exosystem (indirectly affecting environments), macrosystem (cultural and societal context) and chronosystem (temporal context) [8].

Applying Bronfenbrenner’s framework aids to understanding the COVID-19 pandemic’s impact on families [9] on the living system that is a family [10]. This review starts by describing the specific contextual developments that took place during lockdowns in households composed of couples and families with children, living in forced cohabitation.

### The pandemic context

China was the first country to declare confinement in late February 2020, followed by several Asian countries. In Europe, the first localized lockdown took place on late February in Italy, followed by several of other countries in March 2020. In the American continent, localized lockdown also started from the middle of March. Most confinement periods lasted until March 2021.

The pandemic exacerbated pre-existing inequalities in accessing healthcare [11–13], unemployment [14, 15], financial instability [16–19] and confinement stress [20–22].

Families were forced into prolonged cohabitation for long periods [23]. Emotional cohabitation, whether with partners or family of origin, became crucial, as pre-pandemic research indicated complex consequences during catastrophes [24]. Research on the pandemic’s impact on families focused on three areas: (a) identifying risks and challenges [7, 21, 25, 26], (b) factors enabling positive vs. negative adaptation to the crisis [27–30], (c) changes in mental health [31–37] and relationship dynamics [38–43].

The present research follows a similar structure: stressors, processes and outcomes. We start with level one, focusing on stressors–COVID-19 blurred boundaries between home and work [44], as well as limited global travel [45], affecting family and extended family contact, with increased risk of prolonged grief [46] and adolescents [47], as well as parents juggled remote work and homeschooling [45, 48, 49].

The second level explores the processes of family adaptation, that requires a complex interplay between stressors, available resources at individual, relational and family levels, and the family’s appraisal of these stressors [50, 51], influenced by coping models [50–53] and resources [54]. Research with families focused (a) not only on individual models [55] and individual strategies (e.g., problem solving or emotional coping), (b) but also on relational models [56–58] and on the dyadic strategies established by the couple (e.g., problem-focused, or emotion-focused dyadic support), c) and on community resources [59–61].

Finally, the third level encompasses a wide range of outcomes–family members experienced psychopathology [32], such as anxiety [35], depression [36, 37], post-traumatic stress, lower levels of quality of life and general stress [37], increased worry, lack of attention and irritability [31, 33, 34]. Additionally, evaluation of family relations [40–42], in which the literature we find highlights into family resilience with an increase of positive family dynamics [62] to negative consequences [40, 42], ranging from difficulty in eating behaviours or in physical activity [63], to accounts of interpersonal violence [64, 65].

Inconsistencies in data and insufficient literature integration were address by several reviewers, on psychological impact [66] and in specific groups [67]. This study identifies a gap in the literature on COVID-19’s impact on families, focusing on vulnerable households: couples and families in forced cohabitation.

## Method

The present study is a systematic review of the literature that aims to [1] provide an overview of how the COVID-19 pandemic potentially changed family systems, especially the couples and parenting dynamics, as well as [2] integrate inconsistent findings, and, finally, [3] define new avenues for research and clinical practice. To explore the hypothesis of the study quality, we conducted a quality assessment (QuaDS) assessing the theorical framework, methodology, research design and sample characteristics. We undertook a systematic review of the literature that did not focus on meta-analysis results, given the unavailability of data and heterogeneity of design.

### Inclusion and exclusion criteria

Following the systematic reviews guidelines outlined by CRD (2009), articles must satisfy the following inclusion criteria to qualify for this review: (a) published in English, Spanish, Italian or Portuguese, because it is the languages known by the research team; (b) published in a journal with peer review; (c) designed as empirical studies (either quantitative, qualitative or mixed methods); (d) targeting the impact of COVID-19 pandemic on couples functioning with different-gender couples or same-gender couples, married or living together; or with cohabitating parents (at least 90% of the sample) with children between 2 and 18 years old and (f) published between April 2020 to December 2023. The study excluded four types of studies: (a) those involving parents of children with chronic illnesses or prior conditions, due to the added stressors and unique dynamics; (b) parents of newborns born during the COVID-19 pandemic, as this does not align with the aim of comprehending the transition to parenthood; (c) studies with less than 10 participants, to ensure the accuracy and validity of data and its accuracy; and (d) studies that investigate the outcomes of family or couple therapy, as the aim is not to investigate the seeking of professional help by families and couples.

### Procedure

To identify relevant literature for our review, we conducted a systematic literature search across four databases following the guidelines of the Preferred Reporting Items for Systematic Reviews and Meta-Analyses (PRISMA) (2020) [68–70] statement and the Centre for Reviews and Dissemination (CRD) (2009) protocol [71, 72]. Various strategies were utilized to retrieve applicable studies, ensuring a comprehensive review of the literature. First, a computerized search of bibliographic databases PubMed, EBSCO, Google Scholar, and Web of Science was conducted using the following search combinations for the title and abstract: "Couples" AND "COVID-19 Pandemic"; "Romantic Relationships" AND "COVID-19 Pandemic"; "Marital Satisfaction" AND "COVID-19 Pandemic"; "Parental Functioning" AND "COVID-19 Pandemic"; "Parenting" AND "COVID-19 Pandemic"; "Family" AND "COVID-19 Pandemic". In this review, specific journals were not directly consulted for literature related to the review. Instead, we utilized comprehensive databases described above to ensure a wide-ranging and unbiased collection of relevant studies.

A search query was conducted in the Web of Science (Clarivate, 2022) online database to identify relevant studies. This decision was based on a two-step approach. First, a review of similar literature revealed that using multiple databases is the most common practice to ensure comprehensive topic coverage. Second, during the query design phase, extensive trial tests were performed on both Scopus and Web of Science. Web of science consistently produced results that better matched our query criteria, making it a more suitable choice for our review. Therefore, we decided to focus exclusively on Web of science.

### Study selection: Decision marking

The first step on the decision making involved entering the keywords into online data bases. Before screening, we removed the duplicate records. Subsequently, the titles of the articles were reviewed and based on their relevance, they were either advanced to the next stage or excluded. Following this title-based selection, the abstracts were thoroughly read to assess whether they met the inclusion criteria of our systematic review. After this dual stage screening process, which involved both title and abstract evaluation, the remaining articles were full to ensure they satisfied all the inclusion criteria for the study (*See Fig 1 in S1 Fig*). Articles were removed if including having less than 90% of couples living together or having divorced parents. Fifty-eight articles were selected for final comprehensive analysis after a careful review of all remaining articles and analysis of their full texts (*See Fig 1 in S1 Fig*).

### Data collection process

Data extraction was performed by reviewing general study information (author name, article title, citation, type of publication, geographical area, and funding source), study characteristics (aim/objectives of the study, study design, study inclusion and exclusion criteria and recruitment procedures) and participant characteristics (age, gender, ethnicity, marital and cohabitation status, number of children). The method and instruments of each study were analysed for accuracy. After selecting the final articles, authors carefully reviewed the selected and relevant articles, extracted the data, and organised the relevant information. The final evidence table is presented in Table 1 - in this section, we present the author’s name and year of publication, the number of participants in each study, the study design, the geographical area, the data collection time and the main findings.

**Table 1:** Summary of baseline characteristics and outcomes of included studies.

Authors/Year	Country	Sample	Data Collection	Study Design	Main Findings
*Ascigil et al*. *(2023)* ***[73]*** (Reference Number)	USA Canada	*n* = 1873 (Study 1) *n* = 618 (Study 2)	April 2020 (T1) to May 2021 (T6)	Quantitative Study Longitudinal	A variation on relationship satisfaction was found–when conflict and irritation is present in the relationship, satisfaction changes; positive relational processes were associated with prior week satisfaction; high relationship satisfaction was consistently linked to factors such as feeling appreciative and being satisfied with partner quality time at the baseline. Dyadic coping played a more significant role in prior week relationship satisfaction.
*Banaei et al*. *(2021)* ***[74]***	Iran	*n* = 317	April 1st to April 20th, 2020	Quantitative Study Cross-Sectional	COVID-19 pandemic had a significant impact on marital satisfaction. Sexual satisfaction, physical symptoms, anxiety and insomnia, social dysfunction, and depression can significantly affect marital satisfaction during the COVID-19 pandemic.
*Bar-Kalifa et al*. *(2022)* ***[75]***	Israel	*n* = 144	April 30th to June 2nd, 2020	Quantitative Study Cross-Sectional	Stress related to COVID-19 pandemic is associated with lower daily positive mood and higher negative mood. Negative dyadic coping (DC) was associated with lower relational satisfaction and positive DC had a direct effect on relational outcomes. Also, negative DC intensified negative humour and it was found to be more consequential to relational outcomes than positive DC.
*Bar-Shachar et al*. *(2022)* ***[76]***	Israel	*n* = 239	April 12th and April 27th, 2020	Quantitative Study Cross Sectional	Higher levels of relationship satisfaction were associated with increased support and reduced negative behaviours. No significant differences were found before and after covid-19 lockdown. Women reported higher levels of perceived partner support behaviours. Both attachment orientations were associated with lower relationship satisfaction
*Bretaña et al*. *(2023)* ***[77]***	Spain	*n* = 549	10th April and 20th April, 2020	Quantitative Study Cross Sectional	Avoidantly attached individuals’ relationship satisfaction was affected during the confinement, this decrease being explained by strategies used by them and perceived also in their partners during the conflict. Our study has also shed light on the association between some protective factors, like the role of positive problem-solving in relationship satisfaction.
*Budiartini (2021)* ***[78]***	Indonesia	*n* = 242	October to November, 2020	Quantitative Study Cross-Sectional	COVID-19 stress did not affect the marital quality in Bali.
*Carlson et al*. *(2020)* ***[79]***	United States of America (USA)	*n* = 1025	April 2020	Quantitative Study Cross-Sectional	There was an overall increase in domestic responsibilities for mothers, as well as an increase in fathers’ contributions. Both mothers and fathers report a general shift toward more egalitarian divisions of household labor.
*Carvalho & Matias (2023)* ***[80]***	Portugal	*n* = 210	April to June 2020	Quantitative Study Cross Sectional	The study revealed that although the levels of parental exhaustion and relationship quality were not excessively high, there were indications that parental exhaustion contributed to a decrease in relationship satisfaction and an increase in conflict. It is worth noting that positive forms of dyadic coping were identified as moderators, mitigating only the negative impacts on conflict frequency. Couple relationships as being characterized by medium levels of conflict frequency and above average levels of relationship satisfaction.
*Chakraborty et al*. *(2020)* ***[81]***	India	*n* = 119 *n* = 12	April to July 2020	Mixed-Method Study Cross-Sectional	Psychological distress impacted negatively relational quality in terms of conflict, criticism, pressure, dominance, resentment, exclusion, and relative power. The positive impact of psychological distress was observed in terms of affection, emotional support, reliable alliance, satisfaction, approval, and companionship. In terms of psycho-social strategies couples identified conflict resolution, self-introspection, inter-communication improvement and developed a sense of obligation. Also, professional help and recognizing stressors and plan for action as environmental strategies.
*Chung et al*. *(2020)* ***[82]***	Singapore	*n* = 258	22nd April to 5th, 2020	Quantitative Study Cross Sectional	The study revealed that parenting stress played a significant mediating role in the relationship between the perceived impact of COVID-19 and parent-child closeness, as well as harsh parenting. The impact of COVID-19 and stay-at-home orders was linked to increased parenting stress, which, in turn, had a negative effect on parenting. This impact was observed through changes in the way parents interacted with their children, including an increase in the use of harsh parenting techniques.
*Craig & Churchill (2021)* ***[83]***	Australia	*n* = 1536	7th May to 30th May, 2020	Quantitative Study Cross-Sectional	Unpaid work increased and paid work decreased slightly. Mothers did more childcare than fathers, however, fathers contributed more to childcare comparing to before COVID-19 pandemic. More mothers than fathers were dissatisfied with work-family balance and partner’s share comparing to before COVID-19 pandemic.
*Donato et al*. *(2021)* ***[84]***	Italy	*n* = 1823	30th March to 7th April, 2020	Quantitative Study Cross-Sectional	COVID-19 pandemic concerns reflected on poorer psychological well-being (PWB), stress communication and dyadic coping (DC) responses. Stress communication was positively correlated with DC responses but negatively with PWB. DC was positively correlated with PWB. Both satisfied and dissatisfied partners showed similar levels on COVID-19 concerns. Dissatisfied couples showed less explicit stress communication and lower PWB.
*El Akmal et al*. *(2021)* ***[85]***	Indonesia	*n* = 330	Not Available	Quantitative Study Cross-Sectional	Job stress had a negative impact on marital satisfaction and work-life balance had a positive impact of marital satisfaction. Work-life balance influenced marital satisfaction and job stress.
*Feinberg et al*. *(2022)* ***[86]***	USA	*n* = 129	April and May 2020	Quantitative Study Longitudinal	We found large deteriorations from before the pandemic to the first months of the pandemic in child internalizing and externalizing problems and parent depression, and a moderate decline in coparenting quality. Smaller changes were found for parent anxiety and parenting quality. Mothers and families with lower levels of income were at particular risk for deterioration in wellbeing.
*Fleming & Franzese (2021)* ***[87]***	USA	*n* = 782	April 3 to May 22, 2020	Quantitative Study Cross-Sectional	Higher relationship satisfaction during COVID-19 lockdown was related to higher sexual satisfaction, lower relationship invalidation, not having children in the home, higher perceived fairness of relationship power. However, higher thoughts of separation were related to younger age, higher verbal aggression, higher relationship invalidation and lower relationship satisfaction.
*From et al*. *(2023)* ***[88]***	USA Canada	*n* = 600	From April 2020 to May 2021	Quantitative Longitudinal	The study found correlation between similarity in general concern about the pandemic and lower overall relationship quality. Notably, this similarity in concern was also linked to a reduced perception of the pandemic as a source of conflict in the relationship. The participants reported some changes in the relationship satisfaction comparing to before the pandemic.
*Hanetz-Gamliel et al*. *(2021)* ***[89]***	Israel	*n =* 141	Mid-March until the end of April 2020	Quantitative Study Cross-Sectional	Maternal anxiety and hostile parenting behavior mediated the associations between lack of support, negative perceptions about the health and economic threats of COVID-19, and children’ s behavior problems. These findings showed the importance of mothers’ mental health and parenting behaviors for children’ s socioemotional adaptation in the context of COVID-19.
*Hank & Steinback 2021* ***[90]***	Germany	*n =* 3108	Mid-May through early-July 2020	Mixed-Method Study Longitudinal	Results showed that the “traditional” division of housework was the most common arrangement, that decreased 4% during corona virus pandemic. Male contributions increased during the pandemic. The division of labor remained stable in almost 60 percent of the couples. Results showed that when male partner’s working hours increased, the housework done by women also increased (but not childcare). If the men working hours decreased, the female contribution also decreased both in childcare and housework.
*Hiraoka & Tomoda (2020)* ***[91]***	Japan	*n* = 353	29th to 30th April	Quantitative Study Cross-Sectional	There was a significant increase in parenting stress.
*Hood et al*. *(2021)* ***[92]***	Australia	*n* = 30	End of July 2020	Qualitative study Cross-Sectional	Family relationships improved, parental stress increased and there was a reflection on family schedules. Almost a third of mothers described an improved relationship with their children, some participants described changes to their family interactions including more time engaging in home-based family activities. Also, several participants perceived the lockdown as overwhelming and stressful due to uncertainty about the future, having children at home and not being able to leave the house. Many of the mothers reported feeling isolated and alone.
*Hudde et al*. *(2021)* ***[93]***	United Kingdom (UK)	*n* = 6438	April to September 2020	Quantitative Study Longitudinal	Women, especially those with less egalitarian attitudes, spent twice as many hours on housework in some point in time. Women with more egalitarian attitudes reported smaller changes in housework and men with more egalitarian attitudes increased their time spent on housework. Both men and women spent more hours doing housework during COVID-19 pandemic. In all couples, the pandemic increased gender equality in housework in all couples. The authors found no evidence that pre-crisis gender role affected changes in both genders’ contributions to housework during the lockdown.
*Idsøe et al*. *(2021)* ***[94]***	Norway	*n* = 294	Spring, 2020	Quantitative Study Longitudinal	Family activities during lockdown did not impact the pandemic stress levels—9.5% of the mothers and 10.2% of the fathers scored for stress symptoms.
*Jiang et al*. *(2021)* ***[95]***	China	*n* = 108	February to March 2020	Quantitative Study Cross-sectional	Greater daily positive support from their partners is related to greater daily feelings of gratitude and less daily stress during COVID-19 lockdown.
*Jones & Thesis (2021)* ***[96]***	USA	*n* = 302	April through June of 2020	Dyadic Quantitative Study Cross-Sectional	Relational uncertainty due to COVID-19 pandemic and partner interference were positively associated with relational turbulence. The relational turbulence was associated with more severe irritations, more aggressive and less open relational communication, which can deteriorate relationship quality over time.
*Jones et al*. *(2021)* ***[97]***	USA	*n* = 302	April 2020	Qualitative Study Cross-Sectional	A small number of participants reported no effect of COVID-19 on their relationship. Changes in interdependence processes (i.e., establishment of new routines at home, work and relationships; division of household duties and childcare) as well as both positive and negative changes in physical and emotional intimacy. Also, the study reported negative emotions such as feelings of anxiety, uncertainty, tension, irritation, frustration, sadness, depression, and stress. The study also found changes in communication behavior (frequency and quality).
*Karagöz et al*. *(2020)* ***[98]***	Turkey	*n* = 245	May 2020	Quantitative Study Cross-sectional	Sexual function scores during pandemic were lower. Also, anxiety, depression, and stress perception increased during COVID-19 period which also increased sexual dysfunctions in both male and female. Females had more anxiety and depression symptoms and stress perception than males in the pandemic period and were significantly lower during the pandemic than in the preceding period.
*Kolo et al*. *(2021)* ***[99]***	Nigeria	*n* = 10		Qualitative Cross-Sectional	There was a major challenge in the adaptation due to the rapid changes and challenges associated with uncertainty. Participants report the impact in terms of daily routines, home-school, working from home, more household duties, lack of support from day care, changes in sleep, spiritual changes and not being able to visit the loved ones. The study also alights some key-aspects that helped the couples balance work-life: prioritizing, scheduling and time management. The study also reported that it was difficult to balance work-family life due to work activities, stress and mental strains.
*Lee et al*. *(2021)* ***[61]***	USA	*n =* 291	March and April 2020	Quantitative Study Longitudinal	Couples’ disagreement and verbal fighting scores increased from Time 1 to Time 2, but disagreements related to COVID-19 and physical fighting did not. Couples with higher levels of dyadic coping reported fewer fights and disagreements on average. Dyadic coping did not buffer participants from increases in relationship conflict. More days spent in lockdown was associated with increases in disagreements related to COVID-19.
*Li & Samp (2021)* ***[100]***	USA	*n* = 411	April 1st and May 1st, 2020	Quantitative Study Cross-Sectional	Higher perceived threat of COVID-19 predicted more complaint avoidance, which in turn predicted lower relationship satisfaction and higher anxiety, depression, and substance use. Men reported lower relationship satisfaction, more withhold complaints and higher anxiety, depression, and substance use comparing to women. Bisexual individuals reported greater levels of negative pandemic impacts, perceived threat of COVID-19, intentions to terminate the relationships, anxiety, depression, and substance use than lesbian and gay participants. Lesbian participants reported higher relationship satisfaction than other groups.
*McRae (2021)* ***[101]***	New Zealand	*n* = 362	26th March to 28th April, 2020	Quantitative Study Cross-Sectional	During lockdown, parent’s distress predicted an increase on harsh parenting, however, this effect buffered with partner support. Also, predicted a decrease in responsive parenting and parent-child relationship quality which buffered with coparenting. This means that partner support and cooperative coparenting were key-aspects during COVID-19 lockdown to help families navigate stressful and uncertain times.
*Mousavi (2020)* ***[102]***	Iran	*n* = 213	February to mid-April 2020	Mixed-Method Study Cross-Sectional	The effect of lockdown on marital satisfaction and parental burnout was not significant in parents. Fathers had higher psychological well-being than mothers.
*Mutang et al*. *(2022)* ***[103]***	Malasya	*n* = 334	September and December 2020	Quantitative Study Cross Sectional	The study found that participants’ relationship quality improved during the lockdown compared to before the lockdown, namely on commitment, trust, passion, love, and sex components. Stress factors were identified as financial problems, restricted movement, and fear of COVID-19 infection. The stress, depression and anxiety levels were high during the covid-19 lockdown.
*Neff et al*. *(2021)* ***[104]***	USA	*n* = 191	W1 = 16th April to 21st May 2020 W2 = 17th November to 20th December	Quantitative Study Longitudinal	Women, but not men, who were more blaming of the pandemic exhibited reduced stress spillover during the COVID-19 outbreak. Participants were relatively satisfied in their relationship and reported low levels of daily stress.
*Nuru & Bruess (2021)* ***[45]***	USA	*n* = 108	September 2020 to January, 2021	Qualitative Study Cross-Sectional	By engaging in an intentional communicative practice during COVID-19 crisis, couples navigated the COVID-19 pandemic through a dynamic, interwoven processes of negotiating, cultivating, accepting, and inviting (re)construction of their relationship cultures.
*Omar et al*. *(2021)* ***[105]***	Egypt	*n* = 696	30th March to 30th June, 2020	Quantitative Study Cross-Sectional	COVID-19 pandemic was associated with lower sexual satisfaction in both genders. Females however, suffered more anxiety and depression and thereby greater risk of sexual function difficulties and sexual dissatisfaction. Sexual Dynamics Psychological Well-Being
*Osur et al*. *(2021)* ***[106]***	Kenya	*n* = 194	15th to 30th September, 2020	Quantitative Study Cross-Sectional	Sexual satisfaction during the COVID-19 pandemic decreased.
Özlü *et al*. *(2021)* ***[107]***	Turkey	*n* = 329	May and June 2020	Quantitative Study Cross-Sectional	The frequency of sexual intercourse during COVID-19 pandemic decreased for both men and women. Participants had a moderate level of quality of sexual life.
*Panzeri et al*. *(2020)* ***[108]***	Italy	*n* = 124	11th April to 5th May 2020	Quantitative Study Cross-Sectional	Most couples did not perceive any differences in their sexuality. Some female participants reported a decrease in sexual satisfaction, pleasure, sexual desire and arousal because of worry, lack of privacy and stress. Also, participants experienced more anxiety, depression, and stress. This means that personal emotions and psychological difficulties during the lockdown had an impact on participants’ sexual life, more than specific aspects related to the couple’s relationship.
*Partington et al*. *(2022)* ***[109]***	USA	*n* = 449	September and October 2020	Quantitative Study Cross Sectional	This study found that 28% of the families struggled during COVID-19 pandemic and that 15% were distressed during this period. The remaining families were satisfied and adapted to this crisis. Family’s adaptation during COVID-19 changed according to (1) financial background; (2) partner’s contribution to household tasks; (3) coping strategies such as cognitive appraisal and (4) child’s temperament. These findings underscore the multidimensional nature of coping and well-being during COVID-19.
*Quezada Berumen et al*. *(2020)* ***[110]***	México	*n* = 101	18th May to 25th May, 2020	Quantitative Study Cross-Sectional	The higher marital satisfaction the less impact during COVID-19 lockdown in several areas of the person’s life, such as tranquility, happiness, health, physical condition, and emotional well-being.
*Rodríguez-Domínguez (2021)* ***[111]***	Spain	*n =* 342	April 14th to 29th, 2020	Quantitative Study Cross-Sectional	Significant levels of anxiety were associated with poorer dyadic adjustment and a decrease in the perceived quality of relationships since the start of lockdown. Also, increased partner conflict seems to be an important predictor of dyadic adjustment and relationship quality during social isolation. Also, this study suggested that the pandemic had negatively affect the mental health of the population, in particular women.
*Schmid et al*. *(2021)* ***[112]***	Germany	*n* = 781	Mid-May through early July 2020.	Quantitative Study Cross-Sectional	40% of the participants report relationship satisfaction decrease for men and women, 20% increase in relationship satisfaction and 40% remained stable in relationship satisfaction.
*Sels et al*. *(2022)* ***[113]***	Belgian	*n* = 679	From May to August 2020	Cross sectional Quantitative	Parental status was the primary influence on individual and relational well-being, with no significant differences based on gender or sexual orientation. Non-parents reported higher relational well-being than parents, while individuals without children reported a greater increase in perceived depression. Longer-term relationships were associated with lower relational well-being, although this relationship was explained by other factors.
*Seok et al*. *(2021)* ***[114]***	Malaysia	*n* = 124	Not Available	Quantitative Study Cross-Sectional	No significant differences in relationship quality on the couples before and during quarantine except higher trust sub-scale. The Couples that demonstrated lower satisfaction toward their spouses also showed higher commitment, passion, and love in their relationship.
*Shockley et al*. *(2021)* ***[115]***	USA	*n* = 668	T1 = 18th– 23rd March 2020 T2 = 7th– 18th May 2020	Quantitative Study Longitudinal	36.6% of the sample were using strategies in which women did more or all childcare, 18.9% not gendered and 44.5% of the sample used egalitarian strategies. Also, when women did more childcare reported lower well-being and performance at work. Lastly, results show that egalitarian strategies best preserved wives and husband well-being and allowed both to maintain good job performance.
*Soares et al*. *(2021)* ***[65]***	USA	*n* = 1799	30th April– 26th May	Quantitative Study Cross-Sectional	Women spent more time on household duties and childcare during COVID-19 pandemic than men.
*James et al*. *(2022)* ***[116]***	USA	*n* = 734	September and October of 2020	Quantitative Study Cross sectional	There was a decline in relationship satisfaction compared to pre-pandemic reports. This decrease was more pronounced among white individuals, women, less involved parents, and those with higher levels of depressive symptoms. Higher relationship satisfaction was associated with greater support for family policy, particularly in men. At higher levels of relationship satisfaction, men and women showed similar levels of support for family policy. However, at lower levels, women exhibited notably higher support. These findings suggest that the COVID-19 pandemic may have worsened pre-existing social inequalities, particularly those related to significant socioeconomic disparities.
*Spinelli et al*. *(2020)* ***[117]***	Italy	*n* = 810	2nd– 7th April, 2020	Quantitative Study Longitudinal	Household chaos predicted higher levels of parenting stress, which was associated with less effective emotion regulation in children through the mediating role of parental involvement.
*Tan (2021)* ***[118]***	Singapore	*n* = 409	May 2020 (T1) June 2020 (T2)	Quantitative Study Longitudinal	Compared to pre-pandemic levels, the proportion of participants not having marital sex within a week remained stable while weekly sexual frequency increased, with more evenly distributed sexual activity on weekdays and weekends. Stress, fatigue, and marital satisfaction levels predicted probability of non-activity and sexual frequency.
*Tomohiro (2021)* ***[119]***	Japan	*n* = 4882	April, May, August, and December 2020	Quantitative Study Longitudinal	Participants working from home, childcare and household duties increased significantly regardless the gender. There was a decrease on household duties and childcare hours in December 2020 comparing to lockdown period. Increases in household duties, childcare and leisure hours were observed among those who continued to work from home in December 2020 compared to their pre-pandemic levels.
*Turliuc & Candel (2021)* ***[120]***	Romany	*n* = 144	T1 – 16th March,2020 T2 – 15th May, 2020	Quantitative Study Longitudinal	Higher levels of external stress were associated with lower marital satisfaction for women with higher socioeconomic status (SES).
*Vowels et al*. *(2021)* ***[121]***	UK	*n* = 200	March 30th, 2020, and April 21st, 2020,	Mixed-Method Study Longitudinal	Partner’s support was important to thrive during COVID-19 pandemic. Higher relational support predicted better goal outcomes. The qualitative analyses revealed partners use direct and indirect forms of emotional and instrumental support toward goal pursuit.
*Waddell et al*. *(2021)* ***[122]***	New Zealand	*n* = 314	8th to 27th April, 2020	Quantitative Study Longitudinal	Women did more of the parenting and housework that increased relationship problems and decreased relationship satisfaction. Men engaged in more paid work and personal time during the lockdown. Both men and women equally perceived that the relative labor in these domains was unfair.
*Weber et al*. *(2020)* ***[123]***	USA	*n* = 332	2nd May to 11th May, 2020	Quantitative Study Cross-Sectional	In general couples functioning remained the same or improved comparing before COVID-19 pandemic. Also, communication improved while fun in the relationships decreased.
*Williamson (2020)* ***[124]***	USA	*n* = 654	December 2019, March, and April 2020	Quantitative Study Longitudinal	Relationship satisfaction and causal attributions did not change over time, but responsibility attributions decreased on average. There were small moderation effects of relationship coping and conflict during the pandemic, revealing that satisfaction increased, and maladaptive attributions decreased in couples with more positive functioning and satisfaction decreased and maladaptive attributions increased in couples with lower functioning.
*Wong et al*. *(2022)* ***[125]***	USA	*n* = 1642	September 2020 and January 2021	Longitudinal Quantitative	66% of participants reported no change in the quality of their relationships during the pandemic. 22.8% noted an improvement, while 10.5% reported a decline.
*Zamarro & Prados (2021)* ***[126]***	USA	*n* = 3980	10th March to 22nd July, 2020	Quantitative Study Longitudinal	Women spent more time than men in the provision of childcare during the COVID-19 crisis, even while still working. Also, psychological distress emerged in mothers and women without school-age children in the household in early April.
*Zhang et al*. *(2021)* ***[127]***	China	*n* = 1139	March, 2020	Quantitative Study Cross-Sectional	Most of the participants (69.7%) reported no changes on sexual intercourse, sex lives or emotional bonding during COVID-19 pandemic.

## Results

### Overall mapping of the papers

We start this section by describing an outline map of the overall findings with respect to study methodology, location, and article quality. As for the design of the research, quantitative studies were the majority, but qualitative studies, and mixed-method studies were also included (*See Table 1*). Of the quantitative studies, the majority were cross-sectional, and few were longitudinal. As for the qualitative studies, all were cross-sectional [45, 92, 97, 99] and the data collection instruments used were mostly individual semi-structured interviews [92, 99], dyadic interviews [45] and open-ended questions [97]. Regarding mixed-method studies, on the qualitative section some authors used individual semi-structured interviews [90, 102, 121] and on the quantitative section questionnaires were the most common instruments [81, 90, 102, 121]. Studies were longitudinal [90, 121] and were cross-sectional [81, 102].

According to geographical distribution, most studies were conducted in Asia (*n* = 19) and America (*n* = 20), followed by Europe (*n* = 13), Oceania (*n* = 4) and Africa (*n* = 2). The total number of participants across all the studies were *n* = 45572.

### Quality assessment results

Due to the novelty of couple and family research in association with COVID-19, studies were still scattered and showed great heterogeneity in terms of study design. Therefore, the Quality Assessment with Diverse Studies (QuADS) [128, 129] was applied to prevent bias and provide a clear indication of the strength of evidence and standards for future research. Studies were not excluded from this review based on quality, as there were no predefined inclusion and exclusion criteria. All studies were reported, and quality was assessed using 13 items and graded from zero to three.

Following Harrison and colleagues’ (2021) guidelines [128], the quality analysis of the articles was conducted in two phases. In the first phase, two authors performed an individual and independent quality analysis of the studies (*n* = 43) (*See S1–S3 Tables*). In this phase, the authors first selected five studies to perform the quality assessment independently and discussed the application of criteria. Once a common understanding was reached, authors independently reviewed all remaining articles. As the levels of agreement were found to be high, in a second phase, only the main author conducted the quality analysis of the studies (*n* = 15). The final percentage of study quality is presented, ranging from low quality to very good quality (See S1–S3 Tables).

Articles were analysed through NVIVO12, and 13 codes were identified, corresponding to each item and the subcodes according to the score (for example, if an article scored 1 on item 5, it would go to the subcode identified). Articles were evaluated as demonstrating very good (75% to 100%), good (50–75%), medium (25% to 50%) and low quality (0 to 25%). Finally, an interrater analysis was carried on using percent agreement index [129] which showed 91.48% of ratings between the two authors was the same. Most of the studies were classified as good, ten as very good and six as medium (See S1–S3 Tables).

### Thematic analysis results

A qualitative analysis was conducted to identify main themes from key findings in the reviewed articles, following Braun and Clarke’s (2006) thematic analysis approach [130]. In a first moment, data was analysed through hierarchical organisation of initial codes. Initial codes were created inspired by Bronfenbrenner’s Ecological Systems Theory [8], depicting the systems affected by Covid-19—microsystem, mesosystem, macrosystem, exosystem and chronosystem. We also integrated the Stress Model Process [51] in the organization of themes, based on the three elements of stress: 1) sources, 2) mediators, and 3) outcomes. Each code was subdivided and organised into sub-codes. During the second round of data analysis, greater flexibility was enabled in generating the codes using open coding [131, 132]. This involves generating categories in an open and line-by-line manner based on the data, whilst constantly questioning the information presented [132]. After modifying the codes, an inter-categorical analysis was initiated, followed by axial coding involving continuous comparison between categories [132]. Furthermore, the interactions between category dimensions were explored, resulting in the renaming, and identification of new relationships, and even new categories. We also constructed cognitive maps to categorise and formulate new connections that became apparent. Ultimately, the systematic coding facilitated the identification of a range of categories and subcategories regarding the data, to address the research inquiries.

The thorough literature review allowed a categorization of the key domains that appear to have influenced parenting and couples functioning during the COVID-19 pandemic. Nine categories were identified through the thematic analysis. The classification of stress themes based on their sources, mediators, outcomes, and the corresponding systems in which they occurred (*See S4 Table*). In the following section, we describe the themes organized by stress elements, beginning with the themes that arose as stress sources. Subsequently, we outline the themes that acted as mediators and finally, the themes that surfaced as outcomes.

### Themes on stress sources as result of COVID-19 pandemic

Two stress sources categories were identified—Gender Inequalities (*n* = 14) and External Stress (*n* = 15). The Gender Inequalities category is within the macrosystem of the couple, affected by domestic and parental duties and social norms, including two subcategories. The first subcategory named Unequal Division of Household Duties (*n* = 11), refers to an unequal sharing of household management tasks and parental responsibilities in providing primary care for children. The second subcategory named Lack of Support in Maternity (*n* = 3), defined as the absence of support, particularly with regards to childcare and social isolation. Regarding the External Stress (*n* = 15), emerged as well in the macrosystem and is known as the emotional strain and mental tension experienced by couples and parents in adverse and demanding circumstances such as the COVID-19 pandemic. The categories are detailed below.

### Unequal division of household duties (*n* = 11)

The distribution of household duties during the COVID-19 pandemic was evaluated in eleven studies [65, 79, 83, 90, 93, 97, 99, 115, 119, 122, 126]. Since the onset of the COVID-19 pandemic crisis, a noticeable change in the division of housework and childcare [99] was observed. On the one hand, one of the emerging points within this category, as indicated by seven studies, was the gender differences between men and women in the allocation of household work during the COVID-19 outbreak [65, 79, 83, 90, 93, 115, 126]. While there was an increase in fathers’ involvement in domestic tasks, particularly in task allocation, mothers continued to provide more housework and childcare than fathers [65, 79, 83, 90, 93, 115, 122, 126]. The overwhelming domestic and parental burden, in both genders, was reflected in reduced satisfaction [126] and well-being, as well as in work performance [115]—We found an impact of working remotely [119] in family dynamics [99].

### Lack of support for mothers (*n* = 3)

The lack of support for mothers during the pandemic led to feelings of loneliness, isolation, and inadequate support [89, 92, 99]. On one hand, the significance of support systems in parenting practices was evident [92, 99] and on the other hand, a link was observed with more hostile parenting practices and more negative attitudes towards the pandemic [89].

### External stress (*n* = 15)

Elevated stress levels were identified and had a negative impact in dyadic dynamics [75, 81, 89, 91, 92, 94, 99, 101, 104, 117, 120], such as a decline in daily positive mood and an upswing in negative mood amongst couples [75]. The consequences of external stress were not exclusive to couples, as we will mention in the section after.

Focusing on parental stress [80, 82, 86, 89, 91, 92, 101, 109, 117], we identified some contributing factors: (a) household disorganization and chaos [117] and (b) the perception of the pandemic as overwhelming and burdensome [92]. The consequence of parental stress includes (a) a reduction on engagement in their children’s activities; (b) less attention and fewer time spent together; (c) more harsh parenting [82]; (d) less responsive parenting [117] and (e) more conflict in the intimate relationship [80]–those results in a decline in emotional regulation among children [86, 117], more work-family conflict and less balance [99] and parent depression [86].

### Themes on the mediators during COVID-19 pandemic

Coping Strategies *(n* = 21) emerged in the mesosystem, as dyadic coping skills, such as Dyadic Support (*n* = 14), Communication as Mediator (*n* = 4) and Adaptation to New Daily Routines (*n* = 3). Dyadic Support is characterised by one partner helping the other to cope with and overcome problems or challenges, including those caused by COVID-19. Communication as a Mediator (*n* = 3) is defined as the process by which couples send and receive information, verbal or non-verbal, within the relationship. Adaptation to New Daily Routines (*n* = 3), that describes the main day-to-day changes and transformations in couple and parenting dynamics during the COVID-19 pandemic.

### Coping strategies (*n* = 21)

As for the coping mechanisms recognized in the themes, we will first consider dyadic support, before turning our attention to communication and finally to strategies to adapt to new routines imposed by the COVID-19 pandemic.

### Dyadic support (*n* = 14)

Dyadic support was considered a pivotal element in aiding families to cope with the demanding and precarious circumstances posed by the COVID-19 pandemic [111] whilst also proving to be vital for personal development throughout the pandemic period [121]. On the one hand, positive dyadic support reflected on less dyadic conflicts [61, 80], more relationship satisfaction [73, 76, 77, 124], increased gratitude within the relationship, and on more quality in the relationship through the reduction of reducing anxiety levels caused by confinement [95, 111], promoting psychological well-being [75]. On the contrary, negative dyadic coping had a negative impact on relational satisfaction [75, 91, 120] as it accentuated negative mood and had a greater impact on relationships compared to positive dyadic coping [84].

### Communication as mediator (*n* = 3)

Communication emerged as pivotal for couples to handle challenges during the COVID-19 and as a tactic to manage conflict resolution [81, 85], defined as an inter-communication as a psychosocial strategy during conflict management, such as allocating sufficient time for enjoyable active learning [81], negotiating, accepting, and nurturing their relationship cultures [45].

### Adaptation to new daily routines (*n* = 3)

Some changes imposed by the COVID-19 pandemic included the establishment of new routines at home [97, 99] as well as a change in family schedules [92]. Jones [97] found changes in dependency processes, such as the establishment of new routines at home. Kolo and colleagues [99] found that participants reported the impact of the COVID-19 pandemic in terms of daily routines, and Hood et al. [92] showed that family schedules changed.

### Themes on outcomes effects because of COVID-19

Focusing on the outcomes from the COVID-19 pandemic, we found Lower Psychological Well-Being (*n* = 20), as particularly salient in the microsystem, as it reflects individual emotional distress. At the mesosystemic level of the couple, categories such as Relationship (In) Satisfaction (*n* = 21), Sexual Functioning (*n* = 10), and Communication as an Outcome (*n* = 4) have emerged. The Relationship (In) Satisfaction is referred interaction patterns exhibited by couples that affect their satisfaction. The Sexual Functioning category represents the perception of the couples on several sexual dynamics, such as frequency, intimacy, desire, satisfaction, arousal, pleasure, and individual practices (e.g., masturbation). Finally, Communication as a Process is defined as a dimension of the couple that was affected by the pandemic in both positive and negative ways. Relational Growth in the Chronosystem (*n* = 4) emerged as the ability of couples to bounce back and surmount challenges and obstacles during the COVID-19 pandemic.

### Lower psychological well-being (*n* = 20)

Several studies demonstrated psychological difficulties during the COVID-19 crisis [65, 61, 74, 81, 84, 86, 89, 97–100, 102, 103, 108, 110, 111, 113–115, 126]. We also found gender differences in individual well-being, with women reporting inferior levels of well-being [65, 74, 98, 102, 108, 111, 126], especially in women with children [86, 102, 115, 126].

Furthermore, it became evident that these dynamics were reflected in relations, sexual dynamics, and parental interactions. Concerning to relationship dynamics, we found a positive relationship between individual well-being and relationship quality [74, 81], particularly among women [74], that highlight the importance of individual well-being in relation to relationship dynamics [110, 114].

Focusing on the interinfluences between personal well-being and sexual dynamics, some studies have found a negative impact of psychological distress on sexual dysfunctions [98], on pleasure, desire, and arousal [108]. Conversely, elevated levels of personal well-being were reflected in the quality, intimacy, and passion within sexual relationships [114]. Finally, some studies found higher maternal anxiety [89], less relational well-being [113], and poorer parenting behaviours, including those that are hostile [89], as well as parenting being the primary influence on individual and relational well-being [113].

### Relationship (In) satisfaction (*n* = 21)

Several studies examined the levels of satisfaction among couples during the COVID-19 pandemic [73, 74, 77–78, 80, 85, 87, 88, 100, 102–104, 110, 112, 114, 116, 122–125], however the results were not consistent. Focusing on the studies that focused on relationship satisfaction before and after the pandemic, some studies suggested that the COVID-19 pandemic did not affect the satisfaction levels of couples [76, 78, 102, 104, 114, 123–125] and three studies found a decline in relationship satisfaction during the COVID-19 crisis [77, 112, 116]. Two studies found that changes in relationship satisfaction that varied according to relational processes [73] and to the perception of the pandemic as a source of conflict in the relationship [88]. Other studies found high levels of marital satisfaction, namely on relationship quality [103], although not comparing the pre- and post-pandemic periods [80, 85, 100, 110]. Others found that couples were moderately satisfied with their relationship [74, 120, 123]. Some factors were identified as moderators: sexual satisfaction, relationship validation, not having children, perception of fairness in the power dynamics on the relationship, social functioning, not having physical or sleep problems [74, 87], high levels of income [120] and doing activities together as a couple [103].

### Sexual functioning (*n* = 10)

Ten studies analysed couples’ sexual experiences during the COVID-19 pandemic [74, 87, 97, 98, 105–108, 118, 127], but focused on different domains. At an individual level, there was an increase in individual sexual practices both in men and women, but higher in men [98].

At the relationship level, we found a positive impact of sexual satisfaction on relational satisfaction during the COVID-19 pandemic [74, 87]. One of the findings indicated a decrease in sexual satisfaction compared to the pre-pandemic period [98, 105, 106], particularly among women [108], because of the lower levels of well-being being associated with a higher risk of sexual difficulties [105, 118]. Factors contributing to moderate levels of sexual satisfaction included lower relationship invalidation, no children in the home and higher perceived fairness of relationship power [87].

In terms of sexual frequency, one study found a decrease [107] and two studies found no differences before and after the pandemic [74, 127] and one study found an increase on weekly sexual frequency [118]. Two studies reported no changes in sexual desire, arousal, and orgasm [108, 127] comparing to before the pandemic.

In terms of quality of sexual life, one study found a moderate level of quality reported in women and it was influenced by age, education of the spouse as well as the sexual frequency before and during the COVID-19 pandemic [107]. The study suggested that couples with university degrees and specifically women whose partner had a university degree had higher levels of quality of sexual life. Men with a nuclear family had higher levels of quality of sexual life.

Concerning intimacy, one study found both positive and negative changes in intimacy, in which participants described having both more and less time to connect [97] and other found no differences on emotional bonding during COVID-19 pandemic [127].

### Communication as an outcome (*n* = 4)

Some studies have reported changes in the quality and frequency of communication in couples and families during the COVID-19 pandemic. On the one hand, two studies found a decrease in the communication quality, in a more and less communicative engagement with the partner, as well as more negative and more positive valence of conversations [97] such as severe irritation and less open and more aggressive communication, affected relationship quality over time [96]. On the other hand, other study found that communication improved during the COVID-19 pandemic [123], possibly due to fewer external demands such as commuting and travelling in couples with children. Another study found that COVID-19 pandemic concerns were reflected in couples’ stress communication, which was positively correlated with dyadic coping responses, but negatively correlated with psychological well-being [84].

### Relational growth (*n* = 4)

During the COVID-19 pandemic, couples engaged in dynamic processes of negotiation and reconstruction [45], highlighting the importance of coping strategies as promoters of resilience, such as creating new rituals to express care towards their partner [45, 81]. One factor contributing to the relationship satisfaction was attributing any relationship issues to external factors [104]. This led to a decrease in spillover processes, resulting in higher levels of marital satisfaction and fewer negative behaviours.

Furthermore, Chakraborty et al. [81] discovered that psychological distress can have a beneficial effect on a couple’s adjustment during a crisis by promoting affection, emotional support, reliable alliance, satisfaction, approval, and companionship, thereby enhancing relational resilience [81]. More importantly, the study determined psycho-social methods for dealing with COVID-19 challenges, such as communication for conflict resolution, offering emotional support, and respecting personal space. Additionally, self-introspection through dialogue and self-realisation, along with inter-communication improvement through active listening and making time for leisure activities, were identified. Finally, Kolo et al. [99] highlighted the importance of prioritisation, scheduling and time management as crucial factors that aided couples in balancing work and life. These findings imply that these elements could offer valuable insights for interventions aimed at supporting couples during a family crisis [99].

### Integrating inconsistent results

Given the aims of this literature review, the inconsistent findings are discussed in terms of the quality of the papers and the moderating variables. There are three categories where we describe inconsistent results, all of which are presented as outcomes of the process and included as part of the mesosystem: Relationship (In) Satisfaction, Sexual Functioning, and Communication as an Outcome.

### Aim 1. Quality assessment

We first started by crossing the studies with different results against the quality matrix. From the studies reporting good or very good quality, we observed that some changes were identified in relationship (in) satisfaction. On the one hand, the studies reporting good quality found no disparities in relationship satisfaction before and after the COVID-19 pandemic (7 out of 8), fewer studies found a decrease in relationship satisfaction (3 out of 3), other studies found high levels of satisfaction (3 out of 5) and finally other studies found moderate levels (2 out of 2). A pattern was identified, with seven studies reporting both good methodological quality and no changes in relationship satisfaction during the pandemic (*See Table 6 in S4 Table).*

Despite different record of the changes on sexual functioning (both positive and negative changes), most of the studies were classified as of good quality. Several dimensions of sexual functioning have been identified, including sexual satisfaction and sexual frequency. We identified a pattern and observed that sexual satisfaction (3 out of 4) decreased during COVID-19, and that sexual frequency did not change (3 out of 5) (*See Table 7 in S4 Table).*

Finally, as to communication as an outcome, a pattern was identified, with the one study reporting a positive impact being of lower quality that the two that reported a decrease in communication ((*See Table 8 in S4 Table*). As observed during the pandemic there was a negative impact on the level of communication.

### Aim 2. Moderators

To further explore underlying factors of the inconsistent of findings as to couple (in) satisfaction, sexual satisfaction, and communication as an output we will consider two possible hidden moderators. Considering social reports on the economic impact [133, 134] of the COVID-19 pandemic, we will explore the moderating effect of income—a macro variable of the pandemic—on these three categories of mesosystem outcomes. We will also consider the role of coping as a crucial process for crisis intervention at a secondary level.

As to relationship satisfaction, we identified several papers that have information of family income, coping strategies, or both (*See Table 9 in S4 Table*). We found no clear contribution of income in explaining variations in relationship satisfaction when couples were exposed to COVID-19 pandemic stress–some studies reported that income did not moderate slopes in relationship satisfaction [124], others identified financial problems as a stress factor [103], other did not report any changes in income during the pandemic [74, 80, 125] and further reported high- or average-income families [73, 102, 104]. Moving to our second moderator, seven studies showed that coping strategies (positive or negative) do moderate the relationship between pandemic stressors and relationship satisfaction, thus leading to different relationship outcomes. Finally, in three studies that included information on both coping and income [73, 80, 102], we find that the presence of moderate or high income and the development of coping strategies minimized the impact of COVID-19 stress on relationship satisfaction.

Next, we will present the studies with sexual functioning as a dependent variable which refer information on income, coping or both (*See Table 10 in S4 Table).* We found no clear relationship between income and sexual functioning. On the one hand, one study found that low levels of sexual satisfaction were linked to economic losses [98], but other study found no relationship between sexual satisfaction [105] and income. In contrast, moderate levels of sexual satisfaction have been associated with higher income [87]. On the other hand, when no differences in sexual frequency were observed, some studies found that couples tended to have a high income or no changes to the income, and high educational levels [74, 127], and one study found no significant differences [118]. Furthermore, three studies measured both coping, income, and sexual functioning. It appears that the presence of coping strategies and moderate to high income indeed moderated the impact of stress imposed by the pandemic and sexual dynamics [87, 97, 98].

Finally, as to the theme Communication as an Outcome, the only study that have an inconsistent and positive effect of COVID-19 pandemic on communication did not record income and/or coping strategies, so that we cannot move forward in our analysis (*See Table 11 in S4 Table).*

## Discussion

To our knowledge, this is the first systematic review to explore the impact of Covid-19 pandemic on couples and parenting worldwide. From 58 studies across the world, there were 12 themes that interconnected challenges, coping strategies and the impact on parenting and couples during this moment of crisis.

This systematic review of the literature aimed to 1) describe and synthetize the main changes in individual, relationship, and family dynamics during the pandemic; 2) integrate the inconsistent findings and 3) define new routes for research and clinical practice.

Regarding our first goal (to describe the existing thematizes on couples and parenting dynamics during the COVID-19 pandemic), we identified Gender Inequalities and Lack of Support for Mothers relating to how domestic and childcare tasks are divided. This is of particular importance because gender inequalities arise from traditional family models that are structured and operate according to gender roles. This overload of domestic work for women has been described in the literature [135, 136], as a “gender care gap” [137], with repercussions not only on individual well-being [138, 139] but also on the quality of their relationships [140]. We suggest that clinicians should systematically address the division of labour and work-family balance, emphasizing that equitable sharing of responsibilities and improving communication can enhance relationship satisfaction. Additionally, it is important to develop appropriate interventions for mothers, who are a vulnerable group, to address the different dimensions of their overload and risk of mental health issues. This can be achieved by creating programs that offer parental support and reduce psychological distress, hopefully mitigating some of the negative effects of gender inequality. Future investigations should focus on the intersectionality of gender with other factors such as race, socioeconomic status, and single parenthood during crises to understand the unique challenges faced by these groups. This finding also highlights the urgency for the development of more effective support systems, including mental health services, workplace policies, social support networks and community resources. This multifaceted approach advocates for systemic change by targeting policy changes and promoting cultural shifts.

In addition to the stress caused by gender inequalities, external challenges led to high levels of stress in couples and families. Stress can corrode or strengthen relational bonds [141], depending on the resources available [142], on the perception of the situation and on the stress factors [143], that reflect either on maladaptation or relational growth and resilience [143]. In this sense, dyadic support is a significant and relevant aspect highlighted in this review due to existing reduced levels of wellbeing among participants. With effective resources, a period of crisis might carry the potential for positive transformation and present an opportunity for improvement in couples, by building resilience, problem-solving and adaptive skills. Couples can use a crisis as a catalyst for positive change and as a chance to address relational challenges. We believe that the interaction between stress, gender inequalities, and external challenges (e.g., the COVID-19 pandemic) within families depends on the resources available within the dyad. The outcome of this interaction is influenced by how effectively these resources are utilized.

Intimate relationships serve as a context for emotional support and space to alleviate psychological distress, consequently promoting mental and physical health for both family members and couples [144–146]. Also, it is considered a crucial mechanism for overcoming challenges [147] aiding in managing and dealing with stressful situations in a dynamic and reciprocal process [51]. By employing positive coping strategies, relational growth was achieved. We consider that family therapists and other family interventors focus on positive communication skills and assist couples in building an emotionally supportive relational context, which involves both support and stress communication. Since dyadic support becomes particularly important during times of stress, we also believe it is crucial to help couples develop stress management techniques that address both relational and individual stress. Additionally, dyadic support often includes problem-solving skills to address conflicts effectively. Relational growth can emerge from crises, such as the COVID-19 pandemic, when couples foster mutual support and recognize their progress together. Acknowledging families as competent is central to reinforcing positive behaviours and utilizing available resources within the family. Furthermore, since dyadic support is a modifiable dimension, it should be a focus of clinical intervention. Accordingly, therapists can work on changing patterns of dyadic support, communication, validation, and emotional regulation during stressful times. As mentioned before, we found low levels of psychological well-being. Extensive studies conducted during the pandemic have also reported psychological difficulties [31, 33–37]. In the absence of coping strategies and dyadic support, we found a bi-directional relationship between psychological difficulties and couple dynamics [148, 149], also described in the academic field, specifically in parenting [150–152] but also in sexuality [153, 154]. Conversely, insufficient relational satisfaction is reflected in an individual’s well-being [149, 155] and relationship dissatisfaction was found to be strongly linked with emotional distress in both genders [156]. A new aspect that comes to light in this analysis is the effect on work and performance, as previously explored in other research studies [157] and during the COVID-19 pandemic [158]. We believe that this result is a reflection on the gender inequalities and parental overload, as we identify gender-specific challenges, discussed before. Political measures include supportive workplace policies by targeting work-family balance to alleviate the pressure on women and mothers.

Concerning our second goal (integrate inconsistent findings), we found conclusive associations were identified between income, relationship satisfaction, sexual satisfaction, sexual frequency, and stress related to COVID-19. Nonetheless, our observations indicated that individuals with moderate or high income, in conjunction with the implementation of effective coping strategies, exhibited a diminished impact of COVID-19-related stress on both relationship satisfaction and sexual functioning. The effect of coping on the relationship between stressors and marital satisfaction, has been described in the literature as a strong predictor of marital satisfaction [159–161] that confirms our findings. The investigations about relationship between sexual functioning and dyadic coping found that positive dyadic coping has a positive effect on sexual outcomes [161–164].

Regarding income, although we did not find any clear relationship, some studies report that low income can reflect on more fluctuations in marital satisfaction [165], as middle-income couples report both more psychological well-being and dyadic adjustment [166], exerting an important role in marital satisfaction [167–169]. Some factors may contribute to the negative impact of low income on marital satisfaction, namely financial stress [170, 171], financial dependency and difficulties in managing daily life. One possible reason for not finding a moderating effect could be that the families in the study samples maintained their income during the pandemic and may have incurred fewer financial expenses due to lockdown measures, which could have reduced the pressure on the families. Additionally, it is important to note that the primary objective of the studies in this review was not to examine the relationship between income, relational variables, and pandemic-related stress. This socioeconomic vulnerability should be target by public policies to develop support for lower-income individuals and make mental health services available. This can be achieved with public health initiatives as well as workplace policies to improve better work-family balance, as described before, that can be beneficial regardless of income. In the future, academic research should examine how variations in income levels affect the efficacy of different coping strategies and their impact on relationship satisfaction and sexual functioning. Understanding these nuances should better inform targeted therapeutic interventions and policy measures.

Focusing on our third aim (to define new avenues for research and clinical practice), we identified relevant key points: 1) it is essential to identify sources of stress and resources available within families (e.g. coping strategies or socio-demographic characteristics); 2) it is crucial to assess and promote equal sharing of household tasks, as this promotes individual, relational and family well-being and adjustment [172] as well as work-family balance; 3) positive dyadic skills play a central role in the adjustment process and promote resilience and 4) individual well-being needs to be assessed and considered in couple and family therapy.

Regarding the methodological findings, our study included few qualitative studies, so we believe that is important to develop more qualitative studies to capture the perceptions, meanings, and construction of the COVID-19 pandemic experience as a couple and parent, using dyadic interviews. Future investigations should focus on the perspective of both members of the couple and parents at different stages of the family cycle, including longitudinal studies–as all the qualitative studies were cross-sectional. Also, dyadic qualitative studies are greatly needed to understand better the strategies developed to manage childcare demands, division of house duties, and work demands and their respective perception of impact on couple and family quality and satisfaction.

From our perspective, caution should be exercised when considering the findings of the current systematic literature review due to certain limitations. Initially, our study’s research criteria were confined to online databases in peer-reviewed journals as we believed that these publications are more trustworthy and have undergone a thorough review process. Consequently, only peer-reviewed articles were incorporated to ensure that the review’s quality was upheld. Secondly, it is important to recognize the findings while bearing in mind that not all studies had equal methodological rigor.

## Conclusion

Couples and parents faced multiple challenges during this worldwide crisis, which varied according to their individual contexts and living conditions, affecting their individual, relational and family well-being. Our review explores the impact of COVID-19 on these dynamics, which are complex and multifaced. With appropriate resources and strategies, families can transform a period of crisis into an opportunity for growth and change. These resources constitute protective factors for the well-being of couples and families, mitigating difficulties and allowing couples to build new meaning, strategies and relational dynamics. It is necessary to reflect on the risk factors (e.g., unequal household duties, low psychological well-being or poor communication) and the urgent need for intervention and prevention in the future contexts of vulnerability and stress. This includes developing communication skills within dyadic support dynamics, establishing self-care routines, prioritizing mental health, and implementing measures to promote gender equality.

Besides identifying the negative and positive impact of COVID-19 pandemic on relational dynamics, we also identify that significant research gaps remain, particularly regarding marginalized groups. This review identifies strategies to help families cope with crises and prevent some of the relational and individual impacts. To our knowledge, this is the first systematic review that mapped the dynamics of cohabitating families during the COVID-19 pandemic. This review may serve to guide professionals intervening with couples and families, as it identified key areas of prevention and intervention.

## Supporting information

S1 Checklist Prisma statement checklist. (DOCX)

S1 Fig **Fig 1.** PRISMA flowchart for study selection.
(PDF)

S1 Table **Table 1.** Quality assessment for selected studies—quality assessment for diverse studies.
(DOCX)

S2 Table **Table 2.** Quality assessment for selected studies—Quality Assessment for Diverse Studies (QuADS) (2 judges).
(DOCX)

S3 Table **Table 3.** Quality assessment judge 1 and Table 4. Quality Assessment Judge 2.
(DOCX)

S4 Table **Table 5.** Thematic analysis categories by elements of stress and systems, Table 6. relationship satisfaction and quality assessment, **Table 7.** Sexual Functioning and Quality Assessment, **Table 8.** Communication as an Outcome and Quality Assessment, Table 9. Income and Coping on Relationship Satisfaction, **Table 10.** Income and Coping on Sexual Functioning and **Table 11.** Income and Coping on Communication as an Outcome.
(DOCX)

S5 Table **Table 12.** Inclusion criteria for studies targeting couples and **Table 13.** Inclusion Criteria for Studies Targeting Parents.
(DOCX)

S6 Table **Table 14.** Excluded reports for the systematic review of the literature.
(DOCX)
