# Supplementary material for: Couples and parenting dynamics during Covid-19 pandemic: A systematic review of the literature
Source: PLoS One. 2025 Feb 18;20(2):e0315417. doi: 10.1371/journal.pone.0315417 (PMC11835339; doi:10.1371/journal.pone.0315417)
Supplement: S2 Table — Table 2. Quality assessment for selected studies—Quality Assessment for Diverse Studies (QuADS) (2 judges). (DOCX) [file pone.0315417.s004.docx]

**Table 2. Quality Assessment for Selected Studies - Quality Assessment for Diverse Studies (QuADS)^[[1]](#footnote-1)^ (2 Judges)**

| **Authors / Year** | **Total Sum of the Items (Judge 1)** | **Total Sum of the Items (Judge 2)** | **Sum Mean of the Items** | **%** | **Judge 1** | **Judge 2** | **Agreement** |
| --- | --- | --- | --- | --- | --- | --- | --- |
| *Akmal et al. (2021)* | 14 | 14 | 14 | 35,89% | 1 | 1 | 1 |
| *Banaei et al. (2021)* | 21 | 21 | 21 | 53,84% | 2 | 2 | 1 |
| *Bar-Kalifa et al. (2021)* | 23 | 23 | 23 | 58,97% | 2 | 2 | 1 |
| *Berumen et al. (2020)* | 19 | 17 | 18 | 46,15% | 1 | 1 | 1 |
| *Budiartini (2021)* | 15 | 17 | 16 | 41,02% | 1 | 1 | 1 |
| *Carlson et al. (2020)* | 23 | 24 | 23,5 | 60,25% | 2 | 2 | 1 |
| *Chakraboty et al. (2020)* | 15 | 18 | 16,5 | 42,30% | 1 | 1 | 1 |
| *Craig & Churchil (2021)* | 18 | 21 | 20,5 | 52,56% | 1 | 2 | 0 |
| *Donato et al. (2021)* | 27 | 25 | 26 | 66,66% | 2 | 2 | 1 |
| *Fleming & Franzese (2021)* | 21 | 25 | 23 | 58,97% | 2 | 2 | 1 |
| *Hanetz-Gamliel et al. (2021)* | 25 | 32 | 28,5 | 73,07% | 2 | 2 | 1 |
| *Hank & Steinbach (2021)* | 22 | 24 | 23 | 58,97% | 2 | 2 | 1 |
| *Hiroka & Tomada (2020)* | 12 | 14 | 13 | 33,33% | 1 | 1 | 1 |
| *Hood et al. (2021)* | 30 | 32 | 31 | 79,48% | 2 | 2 | 1 |
| *Huddee et al. (2021)* | 28 | 27 | 27,5 | 70,51% | 2 | 2 | 1 |
| *Idsoe et al. (2021)* | 22 | 26 | 24 | 61,53% | 2 | 2 | 1 |
| *Jiang et al. (2021)* | 24 | 32 | 28 | 71,79% | 2 | 2 | 1 |
| *Jones & Theiss (2021)* | 27 | 28 | 27,5 | 70,51% | 2 | 2 | 1 |
| *Jones et al. (2021)* | 26 | 26 | 26 | 66,66% | 2 | 2 | 1 |
| *Karagoz et al. (2021)* | 20 | 21 | 20,5 | 52,56% | 2 | 2 | 1 |
| *Kolo et al. (2021)* | 14 | 13 | 13,5 | 34,61% | 1 | 1 | 1 |
| *Lee et al. (2021)* | 24 | 24 | 24 | 61,53% | 2 | 2 | 1 |
| *Li & Samp (2021)* | 24 | 29 | 26,5 | 67,94% | 2 | 2 | 1 |
| *Lucassen et al. (2021)* | 31 | 32 | 31,5 | 80,76% | 2 | 2 | 1 |
| *McRae et al. (2021)* | 26 | 29 | 27,5 | 70,51% | 2 | 2 | 1 |
| *Mousavi (2020)* | 26 | 27 | 26,5 | 67,94% | 2 | 2 | 1 |
| *Neff et al. (2021)* | 25 | 27 | 26 | 66,66% | 2 | 2 | 1 |
| *Nuru & Bruess (2021)* | 27 | 28 | 27,5 | 70,51% | 2 | 2 | 1 |
| *Osur et al. (2021)* | 24 | 29 | 26,5 | 67,94% | 2 | 2 | 1 |
| *Overall et al. (2021)* | 24 | 24 | 24 | 61,53% | 2 | 2 | 1 |
| *Özlü et al. (2021)* | 23 | 25 | 24 | 61,53% | 2 | 2 | 1 |
| *Panzeri et al. (2020)* | 23 | 24 | 23,5 | 60,25% | 2 | 2 | 1 |
| *Rodriguez-Dominguez et al. (2021)* | 28 | 28 | 28 | 71,79% | 2 | 2 | 1 |
| *Schmid et al. (2021)* | 24 | 29 | 26,5 | 67,94% | 2 | 2 | 1 |
| *Seok et al. (2021)* | 18 | 21 | 19,5 | 50% | 1 | 2 | 0 |
| *Shockley et al. (2021)* | 27 | 30 | 28,5 | 73,07% | 2 | 2 | 1 |
| *Soares et al. (2021)* | 20 | 20 | 20 | 51,28% | 2 | 2 | 1 |
| *Spinelli et al. (2020)* | 22 | 22 | 22 | 56,41% | 2 | 2 | 1 |
| *Turliuc & Candel (2021)* | 22 | 27 | 24,5 | 62,82 | 2 | 2 | 1 |
| *Vowels & Carnelley (2020)* | 28 | 28 | 28 | 71,79% | 2 | 2 | 1 |
| *Vowels et al. (2021)* | 32 | 32 | 32 | 82,05% | 2 | 2 | 1 |
| *Waddell et al. (2021)* | 33 | 31 | 32 | 82,05% | 3 | 2 | 0 |
| *Weber et al. (2021)* | 20 | 20 | 20 | 51,28% | 2 | 2 | 1 |
| *Zamaro & Prados (2021)* | 28 | 27 | 27,5 | 70,51% | 2 | 2 | 1 |
| *Zhang et al. (2021)* | 23 | 24 | 23,5 | 60,25% | 2 | 2 | 1 |

1. Percentage Classification: Low (0-25%), Medium (25%-50%), Good (50%-75%) and Very Good (+ 75%). [↑](#footnote-ref-1)
