# Supplementary material for: Couples and parenting dynamics during Covid-19 pandemic: A systematic review of the literature
Source: PLoS One. 2025 Feb 18;20(2):e0315417. doi: 10.1371/journal.pone.0315417 (PMC11835339; doi:10.1371/journal.pone.0315417)
Supplement: S3 Table — Table 3. Quality assessment judge 1 and Table 4. Quality Assessment Judge 2. (DOCX) [file pone.0315417.s005.docx]

**Table 3. Quality Assessment Judge 1^[[1]](#footnote-1)^**

|  | 1 | 2 | 3 | 4 | 5 | 6 | 7 | 8 | 9 | 10 | 11 | 12 | 13 |
| --- | --- | --- | --- | --- | --- | --- | --- | --- | --- | --- | --- | --- | --- |
| Banaei et al. (2021) |  |  |  |  |  |  |  |  |  |  |  |  |  |
| Bar-Kalifa et al. (2021) |  |  |  |  |  |  |  |  |  |  |  |  |  |
| Quezada Berumen et al. (2020) |  |  |  |  |  |  |  |  |  |  |  |  |  |
| Budiartini (2021) |  |  |  |  |  |  |  |  |  |  |  |  |  |
| Carlson et al. (2020) |  |  |  |  |  |  |  |  |  |  |  |  |  |
| Chakraborty et al. (2020) |  |  |  |  |  |  |  |  |  |  |  |  |  |
| Craig & Churchill (2021) |  |  |  |  |  |  |  |  |  |  |  |  |  |
| Donato et al. (2021) |  |  |  |  |  |  |  |  |  |  |  |  |  |
| AkmalEl Akmal et al., 2021 |  |  |  |  |  |  |  |  |  |  |  |  |  |
| Fleming & Franzese (2021) |  |  |  |  |  |  |  |  |  |  |  |  |  |
| Hanetz-Gamliel et al. (2021) |  |  |  |  |  |  |  |  |  |  |  |  |  |
| Hank & Steinback 2021 |  |  |  |  |  |  |  |  |  |  |  |  |  |
| Hiraoka & Tomoda (2020) |  |  |  |  |  |  |  |  |  |  |  |  |  |
| Hood et al. (2021) |  |  |  |  |  |  |  |  |  |  |  |  |  |
| Huddee et al. (2021) |  |  |  |  |  |  |  |  |  |  |  |  |  |
| Idsoe et al. (2021) |  |  |  |  |  |  |  |  |  |  |  |  |  |
| Jiang et al. (2021) |  |  |  |  |  |  |  |  |  |  |  |  |  |
| Jones & Thesis (2021) |  |  |  |  |  |  |  |  |  |  |  |  |  |
| Jones et al. (2021) |  |  |  |  |  |  |  |  |  |  |  |  |  |
| Karagöz et al. (2020) |  |  |  |  |  |  |  |  |  |  |  |  |  |
| Kolo et al. (2021) |  |  |  |  |  |  |  |  |  |  |  |  |  |
| Lee et al. (2021) |  |  |  |  |  |  |  |  |  |  |  |  |  |
| Li & Samp (2021) |  |  |  |  |  |  |  |  |  |  |  |  |  |
| McRae et al., 2021 |  |  |  |  |  |  |  |  |  |  |  |  |  |
| Mousavi (2020) |  |  |  |  |  |  |  |  |  |  |  |  |  |
| Neff et al. (2021) |  |  |  |  |  |  |  |  |  |  |  |  |  |
| Nuru & Bruess (2021) |  |  |  |  |  |  |  |  |  |  |  |  |  |
| Osur et al. (2021) |  |  |  |  |  |  |  |  |  |  |  |  |  |
| Ozlu et al. (2021) |  |  |  |  |  |  |  |  |  |  |  |  |  |
| Panzeri et al. (2020) |  |  |  |  |  |  |  |  |  |  |  |  |  |
| Rodríguez-Domínguez et al. (2021) |  |  |  |  |  |  |  |  |  |  |  |  |  |
| Schmid et al. (2021) |  |  |  |  |  |  |  |  |  |  |  |  |  |
| Seok et al. (2021) |  |  |  |  |  |  |  |  |  |  |  |  |  |
| Shockley et al. (2021) |  |  |  |  |  |  |  |  |  |  |  |  |  |
| Soares et al. (2021) |  |  |  |  |  |  |  |  |  |  |  |  |  |
| Spinelli et al. (2020) |  |  |  |  |  |  |  |  |  |  |  |  |  |
| Turliuc & Candel (2021) |  |  |  |  |  |  |  |  |  |  |  |  |  |
| Vowels & Carnelley 2021 |  |  |  |  |  |  |  |  |  |  |  |  |  |
| Vowels et al. (2021) |  |  |  |  |  |  |  |  |  |  |  |  |  |
| Waddell et al. (2021) |  |  |  |  |  |  |  |  |  |  |  |  |  |
| Weber et al. (2021) |  |  |  |  |  |  |  |  |  |  |  |  |  |
| Zamarro & Prados (2021) |  |  |  |  |  |  |  |  |  |  |  |  |  |
| Zhang et al. (2021) |  |  |  |  |  |  |  |  |  |  |  |  |  |

**Note**: Item Classification: Blue = 0 (not at all), Green=1(very slightly), Yellow=2 (moderately) and 3=Orange

**Table 4. Quality Assessment Judge 2^[[2]](#footnote-2)^**

|  | 1 | 2 | 3 | 4 | 5 | 6 | 7 | 8 | 9 | 10 | 11 | 12 | 13 |
| --- | --- | --- | --- | --- | --- | --- | --- | --- | --- | --- | --- | --- | --- |
| Banaei et al. (2021) |  |  |  |  |  |  |  |  |  |  |  |  |  |
| Bar-Kalifa et al. (2021) |  |  |  |  |  |  |  |  |  |  |  |  |  |
| Quezada Berumen et al. (2020) |  |  |  |  |  |  |  |  |  |  |  |  |  |
| Budiartini (2021) |  |  |  |  |  |  |  |  |  |  |  |  |  |
| Carlson et al. (2020) |  |  |  |  |  |  |  |  |  |  |  |  |  |
| Chakraborty et al. (2020) |  |  |  |  |  |  |  |  |  |  |  |  |  |
| Craig & Churchill (2021) |  |  |  |  |  |  |  |  |  |  |  |  |  |
| Donato et al. (2021) |  |  |  |  |  |  |  |  |  |  |  |  |  |
| AkmalEl Akmal et al., 2021 |  |  |  |  |  |  |  |  |  |  |  |  |  |
| Fleming & Franzese (2021) |  |  |  |  |  |  |  |  |  |  |  |  |  |
| Hanetz-Gamliel et al. (2021) |  |  |  |  |  |  |  |  |  |  |  |  |  |
| Hank & Steinback 2021 |  |  |  |  |  |  |  |  |  |  |  |  |  |
| Hiraoka & Tomoda (2020) |  |  |  |  |  |  |  |  |  |  |  |  |  |
| Hood et al. (2021) |  |  |  |  |  |  |  |  |  |  |  |  |  |
| Huddee et al. (2021) |  |  |  |  |  |  |  |  |  |  |  |  |  |
| Idsoe et al. (2021) |  |  |  |  |  |  |  |  |  |  |  |  |  |
| Jiang et al. (2021) |  |  |  |  |  |  |  |  |  |  |  |  |  |
| Jones & Thesis (2021) |  |  |  |  |  |  |  |  |  |  |  |  |  |
| Jones et al. (2021) |  |  |  |  |  |  |  |  |  |  |  |  |  |
| Karagöz et al. (2020) |  |  |  |  |  |  |  |  |  |  |  |  |  |
| Kolo et al. (2021) |  |  |  |  |  |  |  |  |  |  |  |  |  |
| Lee et al. (2021) |  |  |  |  |  |  |  |  |  |  |  |  |  |
| Li & Samp (2021) |  |  |  |  |  |  |  |  |  |  |  |  |  |
| McRae et al., 2021 |  |  |  |  |  |  |  |  |  |  |  |  |  |
| Mousavi (2020) |  |  |  |  |  |  |  |  |  |  |  |  |  |
| Neff et al. (2021) |  |  |  |  |  |  |  |  |  |  |  |  |  |
| Nuru & Bruess (2021) |  |  |  |  |  |  |  |  |  |  |  |  |  |
| Osur et al. (2021) |  |  |  |  |  |  |  |  |  |  |  |  |  |
| Ozlu et al. (2021) |  |  |  |  |  |  |  |  |  |  |  |  |  |
| Panzeri et al. (2020) |  |  |  |  |  |  |  |  |  |  |  |  |  |
| Rodríguez-Domínguez et al. (2021) |  |  |  |  |  |  |  |  |  |  |  |  |  |
| Schmid et al. (2021) |  |  |  |  |  |  |  |  |  |  |  |  |  |
| Seok et al. (2021) |  |  |  |  |  |  |  |  |  |  |  |  |  |
| Shockley et al. (2021) |  |  |  |  |  |  |  |  |  |  |  |  |  |
| Soares et al. (2021) |  |  |  |  |  |  |  |  |  |  |  |  |  |
| Spinelli et al. (2020) |  |  |  |  |  |  |  |  |  |  |  |  |  |
| Turliuc & Candel (2021) |  |  |  |  |  |  |  |  |  |  |  |  |  |
| Vowels & Carnelley 2021 |  |  |  |  |  |  |  |  |  |  |  |  |  |
| Vowels et al. (2021) |  |  |  |  |  |  |  |  |  |  |  |  |  |
| Waddell et al. (2021) |  |  |  |  |  |  |  |  |  |  |  |  |  |
| Weber et al. (2021) |  |  |  |  |  |  |  |  |  |  |  |  |  |
| Zamarro & Prados (2021) |  |  |  |  |  |  |  |  |  |  |  |  |  |
| Zhang et al. (2021) |  |  |  |  |  |  |  |  |  |  |  |  |  |

**Note**: Item Classification: Blue = 0 (not at all), Green=1(very slightly), Yellow=2 (moderately) and 3=Orange

1. Item 1: Theoretical or conceptual underpinning to the research; Item 2: Statement of research aim(s); Item 3: Clear description of research setting and target population; Item 4:The study design is appropriate to address the stated research aim(s); Item 5: Appropriate sampling to address the research aim(s); Item 6: Rationale for choice of data collection tool(s); Item 7: The format and content of data collection tool is appropriate to address the stated research aim(s); Item 8: Description of data collection procedure; Item 9: Recruitment data provided; Item 10: Justification for analytic method selected; Item 11: The method of analysis was appropriate to answer the research aims; Item 12: Evidence that the research stakeholders have been considered in research design and conduct; Item 13: Strengths and limitations critically discussed. [↑](#footnote-ref-1)
2. Item 1: Theoretical or conceptual underpinning to the research; Item 2: Statement of research aim(s); Item 3: Clear description of research setting and target population; Item 4:The study design is appropriate to address the stated research aim(s); Item 5: Appropriate sampling to address the research aim(s); Item 6: Rationale for choice of data collection tool(s); Item 7: The format and content of data collection tool is appropriate to address the stated research aim(s); Item 8: Description of data collection procedure; Item 9: Recruitment data provided; Item 10: Justification for analytic method selected; Item 11: The method of analysis was appropriate to answer the research aims; Item 12: Evidence that the research stakeholders have been considered in research design and conduct; Item 13: Strengths and limitations critically discussed. [↑](#footnote-ref-2)
