# Supplementary material for: Couples and parenting dynamics during Covid-19 pandemic: A systematic review of the literature
Source: PLoS One. 2025 Feb 18;20(2):e0315417. doi: 10.1371/journal.pone.0315417 (PMC11835339; doi:10.1371/journal.pone.0315417)
Supplement: S4 Table — Table 5. Thematic analysis categories by elements of stress and systems, Table 6. relationship satisfaction and quality assessment, Table 7. Sexual Functioning and Quality Assessment, Table 8. Communication as an Outcome and Quality Assessment, Table 9. Income and Coping on Relationship Satisfaction, Table 10. Income and Coping on Sexual Functioning and Table 11. Income and Coping on Communication as an Outcome. (DOCX) [file pone.0315417.s006.docx]

**Table 5. Thematic Analysis Categories by Elements of Stress and Systems**

|  | **Category** | **System** |
| --- | --- | --- |
| *Sources* | Gender Inequalities | Macrosystem |
|  | External Stress | Macrosystem |
| *Mediator* | Coping Strategies | Mesosystem |
|  | Change in Daily Routines | Mesosystem |
|  | Relationship Growth | Chronosystem |
| *Outcome* | Lower Psychological Well being | Microsystem |
|  | Relationship Satisfaction | Mesosystem |
|  | Sexual Functioning | Mesosystem |
|  | Communication as Outcome | Mesosystem |

**Table 6. Relationship Satisfaction and Quality Assessment**

| **Study** | **Quality Assessment** | **Result** |
| --- | --- | --- |
| *Budiartini (2021)* | Medium | No changes |
| *Mousavi (2020)* | Good | No changes |
| *Neff et al. (2021)* | Good | No changes |
| *Seok et al. (2021)* | Good | No changes |
| *Weber et al. (2021)* | Good | No changes |
| *Williamson (2020)* | Good | No changes |
| *Wong et al. (2022)* | Good | No changes |
| *Bar-Shachar et al. (2022)* | Good | No changes |
| *El Akmal et al. (2021)* | Medium | High levels |
| *Berumen et al. (2020)* | Medium | High levels |
| *Li & Samp (2021)* | Good | High levels |
| *Carvalho & Matias (2023)* | Good | High levels |
| *Mutang et al. (2023)* | Good | High levels |
| *Fleming and Franzese (2021)* | Good | Moderate Levels |
| *Banaei et al. (2021)* | Good | Moderate Levels |
| *Schmid et al. (2021)* | Good | Decreases |
| *Bretaña et al. (2023)* | Good | Decreases |
| *James et al. (2022)* | Very Good | Decreases |
| *Ascigil et al. (2023)* | Very Good | Changes |
| *From et al. (2023)* | Good | Changes |

**Table 7. Sexual Functioning and Quality Assessment**

| **Study** | **Quality Assessment** | **Domain** | **Result** |
| --- | --- | --- | --- |
| *Jones et al. (2021)* | Good | Sexual Intimacy | Both positive and negative changes |
| *Karagoz et al. (2020)* | Good | Individual Practises | Increase |
|  |  | Sexual Satisfaction | Decrease |
| *Panzeri et al. (2020)* | Good | Sexual Desire | No changes |
|  |  | Sexual Frequency | No changes |
| *Zhang et al. (2021)* | Good | Sexual Desire | No changes |
|  |  | Sexual Frequency | No changes |
| *Özlü et al. (2021)* | Good | Sexual Frequency | Decrease |
|  |  | Sexual Quality | Moderate |
| *Osur et al. (2021)* | Good | Sexual Satisfaction | Decrease |
| *Banaei et al. (2020)* | Good | Sexual Frequency | No changes |
|  |  |  |  |
| *Fleming and Franzese (2021)* | Good | Sexual Satisfaction | Moderate |
| *Omar et al. (2022)* | Good | Sexual Satisfaction | Decrease |
| *Tan (2021)* | Very Good | Sexual Frequency | Increase |

**Table 8. Communication as an Outcome and Quality Assessment**

| **Study** | **Quality Assessment** | **Result** |
| --- | --- | --- |
| *Jones et al. (2021)* | Good | Negative Impact |
| *Jones & Thesis (2021)* | Good | Negative Impact |
| *Weber et al. (2021)* | Medium | Positive Impact |

**Table 9. Income and Coping on Relationship Satisfaction**

| **Study** | **Quality Assessment** | **Result** | **Income** | **Coping Strategies** |
| --- | --- | --- | --- | --- |
| *Ascigil et al. (2023)* | Very Good | Changes | High income sample. | Dyadic coping was predictive of prior week relationship satisfaction. |
| *Banaei et al. (2021)* | Good | Moderate Levels | The participants did not have reduced income due to COVID-19 pandemic. | X |
| *Bar-Shachar et al. (2022)* | Good | No Changes | X | Attachment avoidance and anxiety were linked to perceiving reduced support and increased negative responses to COVID-related concerns. Greater satisfaction in relationships was associated with elevated support levels and decreased occurrences of negative behaviours. |
| *Berumen et al. (2020)* | Medium | High levels | X | To deal with the constraints of confinement, couples prioritized upholding a regular routine, including scheduling activities (25.7%), engaging in enjoyable pastimes, such as watching films or TV series (21.8%), and exercising (14.9%) at a higher frequency. Research revealed varying activities that aid in managing the situation of confinement. |
| *Bretaña et al.*  *(2023)* | Good | Decreases | X | Diminished levels of relationship satisfaction can be attributed to individuals' perceptions of the conflict resolution strategies employed by both them and their partners.  Specifically, avoidantly attached individuals experienced a decline in relationship satisfaction during confinement, with this decrease linked to the strategies employed by them and observed in their partners during conflicts. Additionally, the study has illuminated the connection between protective factors, such as the positive role of problem-solving, and overall relationship satisfaction. |
| *Budiartini (2021)* | Medium | No changes | X | X |
| *Carvalho & Matias (2023)* | Good | High Levels | Many individuals (58.1%) had medium household income, and for the majority (68.1%), their financial status did not change after the lockdown | The research discovered elevated levels of dyadic coping. It also identified a positive connection between dyadic coping and relationship satisfaction, along with significant negative associations between dyadic coping and the frequency of conflicts. Dyadic coping was observed to moderate the influence on conflict frequency, but not on relationship satisfaction. |
| *El Akmal et al. (2021)* | Medium | High levels | X | X |
| *Fleming and Franzese (2021)* | Good | Moderate Levels | Actual income loss was not related to satisfaction, while money stress in the relationship was. Although the pure economics of this situation are stressful, those relationships are more affected by how intimate partners respond to the financial issues and work together around them, at least in couples with relatively higher average incomes as seen in this study. | This study suggests that maintaining emotional and physical intimacy during the pandemic can buffer against the negative environment. |
| *From et al. (2023)* | Good | Changes | X | X |
| *James et al. (2022)* | Very Good | Decreases | X | X |
| *Li & Samp (2021)* | Good | High levels | Income was negatively related to the pandemic’s adverse impacts, intentions to end the relationships, anxiety, depression, and substance use. | Complaint avoidance was negatively related to relationship satisfaction, and positively associated with anxiety, depression, and substance use. |
| *Mousavi (2020)* | Good | No changes | Parents residing in average communities and have a paid profession experienced greater well-being compared to parents residing in disadvantaged neighbourhoods | Supportive resources within family-based culture for lessening the detrimental impacts of stressful circumstances on family interactions. Parental involvement in various activities such as art and household chores, establishing virtual connections with family members, reinforcing religious beliefs can considerably mitigate the unfavourable effects of stressful events on family outcomes. |
| *Mutang et al. (2022)* | Good | High Levels | Financial problems were identified as a source of stress in adults during COVID-19 | X |
| *Neff et al. (2021)* | Good | No changes | Individuals’ median income was between US$40,000 and US$49,000 | The research revealed that participants' partners tended to attribute blame for their difficulties to the perceived stressor when they encountered uncontrollable stressors, which appeared to boost their resilience towards stress. |
| *Schmid et al. (2021)* | Good | Decreases | X | X |
| *Seok et al. (2021)* | Good | No changes | X | X |
| *Weber et al. (2021)* | Good | No changes | X | X |
| *Williamson (2020)* | Good | No Changes | Income did not moderate slopes in relationship satisfaction, causal attributions, or responsibility attributions. | Levels of relationship coping and conflict significantly moderated alterations in relationship satisfaction, causal attributions, and responsibility attributions. These moderation effects were of small to medium magnitude. For individuals with higher coping levels, there was an increase in relationship satisfaction and a decrease in causal and responsibility attributions. Conversely, among those with lower coping levels, relationship satisfaction decreased, causal attributions increased, and responsibility attributions remained stable. |
| *Wong et al. (2022)* | Good | No Changes | 18.8% had worse income comparing to before the pandemic; 76.4% same as before and 4.9% a better income. | X |

**Table 10. Income and Coping on Sexual Functioning**

| **Study** | **Quality Assessment** | **Domain** | **Result** | **Income** | **Coping** |
| --- | --- | --- | --- | --- | --- |
| *Jones et al. (2021)* | Good | Sexual Intimacy | Both positive and negative changes | Participants reported household income of $0-$40,000 (24.2%, *n* = 73), $40,001-$80,000 (31.8%, *n* = 96), $80,001-$120,000  (26.8%, *n* = 81), $120,001-$160,000 (12.9%, *n* = 39), and $160,001 or greater (4.3%, *n* = 13). | Reinforcing intimacy and connection was the most cited coping strategy for individuals who identified negative emotions, uncertainty, and stress (19.9%, n= 61) or changes in intimacy (20.3%, n = 99) as sources of turbulence. |
| *Karagoz et al. (2020)* | Good | Individual Practises | Increase | Erectile function domain had significantly negative correlation with economic loss. | The couples that spent more time together during the pandemic reported better sexual function scores. |
|  |  | Sexual Satisfaction | Decrease |  |  |
| *Panzeri et al. (2020)* | Good | Sexual Desire | No changes | X | X |
|  |  | Sexual Frequency | No changes | X | X |
| *Zhang et al. (2021)* | Good | Sexual Desire | No changes | Being in a full-time job was significantly associated with positive sexual health outcomes including quality of sexual life and emotional bonding. Therefore, having a full-time job was more likely to lead to more economic stability and less stress, which could then impact sexuality. | X |
|  |  | Sexual Frequency | No changes |  |  |
| *Özlü et al. (2021)* | Good | Sexual Frequency | Decrease | In the current research, it was observed that both male and female participants with university degrees, as well as female participants with spouses holding university degrees, exhibited elevated levels of sexual life quality. The association may be attributed to the likelihood that individuals with university degrees often secure income-generating jobs, leading to higher income levels and more comfortable living conditions. | X |
|  |  | Sexual Quality | Moderate |  | X |
| *Osur et al. (2021)* | Good | Sexual Satisfaction | Decrease | X | X |
| *Banaei et al. (2020)* | Good | Sexual Frequency | No changes | The participants did not have reduced income due to COVID-19 pandemic. | X |
| *Fleming and Franzese (2021)* | Good | Sexual Satisfaction | Moderate | While the economic aspects of this scenario induce stress, the impact on relationships is more influenced by the way intimate partners handle financial challenges collaboratively, particularly in couples with relatively higher average incomes, as evidenced by the findings in this study. | This study suggests that maintaining emotional and physical intimacy during the pandemic can buffer against the negative environment. |
| *Omar et al. (2020)* | Good | Sexual Satisfaction | Decrease | No relation was found between sexual satisfaction and monthly income. | X |
| *Tan (2021)* | Very Good | Sexual Frequency | Increase | No significant differences were found between income levels and sexual frequency. | X |

**Table 11. Income and Coping on Communication as an Outcome**

| **Study** | **Quality Assessment** | **Result** | **Income** | **Coping Strategies** |
| --- | --- | --- | --- | --- |
| *Jones et al. (2021)* | Good | Negative | Participants reported household income of $0-$40,000 (24.2%, *n* = 73), $40,001-$80,000 (31.8%, *n* = 96), $80,001-$120,000  (26.8%, *n* = 81), $120,001-$160,000 (12.9%, *n* = 39), and $160,001 or greater (4.3%, *n* = 13). | The study found eight coping strategies (a) seeking escape, (b) reinforcing intimacy and connection, (c) managing routines, (d) engaging social networks, (e) practicing mindfulness, (f) purposeful use of time, (g) setting boundaries, and (h) planning for the future. For couples with children, the top three coping strategies were managing routines (27.4%), reinforcing intimacy and connection (23.6%), and seeking escape (16%), whereas for couples without children their top three categories were seeking escape (23%), reinforcing intimacy and connection (19.3%), and managing routines (17.7%).Reinforcing intimacy and connection was the most cited coping strategy for individuals who identified negative emotions, uncertainty, and stress (19.9%, n= 61) or changes in intimacy (20.3%, n = 99) as sources of turbulence. |
| *Jones & Theiss (2021)* | Good | Negative | Participants reported household income of less than $60,000 (39.7% of participants), $60,001 to $120,000 (43%), and more than $120,001 (17.3%). Further, 35.7% of participants reported a loss of household income due to COVID-19, ranging from less than $1000 to more than $10,000. | X |
| *Weber et al. (2021)* | Medium | Positive | X | X |
