# Supplementary material for: Couples and parenting dynamics during Covid-19 pandemic: A systematic review of the literature
Source: PLoS One. 2025 Feb 18;20(2):e0315417. doi: 10.1371/journal.pone.0315417 (PMC11835339; doi:10.1371/journal.pone.0315417)
Supplement: S5 Table — Table 12. Inclusion criteria for studies targeting couples and Table 13. Inclusion Criteria for Studies Targeting Parents. (DOCX) [file pone.0315417.s007.docx]

**Table 12. Inclusion Criteria for Studies Targeting Couples**

| **Date of Publication** | **Language** | **Published in Journal with Peer Review** | **Empirical Study** | **Target of the Study** | **Sample Characteristics** | **Data Collection** |
| --- | --- | --- | --- | --- | --- | --- |
| Ascigil et al. (2023) | English | Yes.  <https://doi.org/10.1111/spc3.12881> | Quantitative Study  Longitudinal | Relationship satisfaction and it’s predictors. | Participants were in a romantic relationship and living together. | April 2020 to May 2021 |
| Banaei et al. (2021) | English | Yes.  <https://doi.org/10.5812/semj.112434> | Quantitative Study  Cross-Sectional | Marital satisfaction in iranian women during  the Covid-19 pandemic. | Participants were married women living with their partners. | April 2020 |
| Bar-Kalifa et al. (2022) | English | Yes.  <https://doi.org/10.1037/emo0000971> | Quantitative Study  Cross-Sectional | Dyadic coping during the Covid-19 pandemic. | Participants were cohabitating couples. | April 30th to June 2nd, 2020 |
| Bar-Shachar et al. (2022) | English | Yes. <https://doi.org/10.1111/fare.12767> | Quantitative Study  Cross Sectional | Relationship satisfaction during the Covid-19 pandemic. | Participants were adult couples who had lived together and been in a relationship for at least 6 months. | April 12th and April 27th, 2020 |
| Bretaña et al. (2023)  (162) | English | Yes.  <https://doi.org/10.1002/pchj.646> | Quantitative Study  Cross Sectional | Conflict resolution and relationship dynamics during the Covid-19 pandemic. | Participants were couples living together. | 10th April and 20th April, 2020 |
| Budiartini (2021)  (110) | English | Yes.  https://[10.21831/pri. v4i1.43287](http://dx.doi.org/10.21831/pri.v4i1.43287) | Quantitative Study  Cross-Sectional | The effect of stress during the pandemic on marital quality during the Covid-19 pandemic. | Participants were married couples living together. | October to November, 2020 |
| Chakraborty et al. (2020)  (74) | English | Yes.  <https://doi.org/10.30877/ijmh.8.2.2021.192-199> | Mixed-Method Study  Cross-Sectional | Psychological distress and couple’s functioning during Covid-19 pandemic. | Participants were all married. | April to July 2020 |
| Craig & Churchill (2021) | English | Yes.  <https://doi.org/10.1111/gwao.12497> | Quantitative Study  Cross-Sectional | Dual-earner, parent and couples’ work and care during the Covid-19 pandemic. | Participants were all living with the partner. | May 2020 |
| Donato et al. (2021) | English | Yes. <https://doi.org/10.3389/fpsyg.2020.578395> | Quantitative Study  Cross-Sectional | Dyadic coping, psychological well-being the Covid-19 pandemic. | Participants were individuals in a couple relationship, married and/or cohabitating. | 30th March to 7th April, 2020 |
| El Akmal et al. (2021) | English | Yes.  ISSN(Online): 2455-9024 | Quantitative Study  Cross-Sectional | Work-life balance, job stress, and marital satisfaction during Covid-19 pandemic. | Participants included married women. | Not Available |
| Fleming & Franzese (2021) | English | Yes. https://doi.org/10.1037/cfp0000169 | Quantitative Study  Cross-Sectional | Predictors of relationship satisfaction during Covid-19 pandemic. | This sample included cohabitating couples. | April 3 to May 22, 2020 |
| From et al. (2023) | English | Yes: https://10.1111/spc3.12789 | Quantitative  Longitudinal | Romantic  relationship quality during Covid-19 pandemic. | In this sample, participants were living with the romantic partner. | From April 2020 to May 2021 |
| Hank & Steinback 2021 | English | Yes.  https://doi.org/10.20377/jfr-488 | Mixed-Method Study  Longitudinal | Division of household duties during Covid-19 pandemic. | In this sample, respondents were cohabiting with a partner. | Mid-May through early-July 2020 |
| Hudde et al. (2021) | English | Yes. https://doi.org/10.1177/23780231211064395 | Quantitative Study  Longitudinal | Housework during  the COVID-19 Pandemic. | Participants were british couples. | April to September 2020 |
| Jiang et al. (2021) | English | Yes. https://doi.org/10.1007/s10902-021-00387-0 | Quantitative Study  Cross-sectional | Dyadic support and stress during Covid-19 pandemic. | Participants were married couples living together. | February to March 2020 |
| Jones & Thesis (2021) | English | Yes. <https://doi.org/10.1177/02654075211044491> | Dyadic Quantitative Study  Cross-Sectional | Relationship outcomes and turbulence theory during the Covid-19 pandemic. | Participants were couples cohabiting with their romantic partner. | April through June of 2020 |
| Jones et al. (2021) | English | Yes. https://doi.org/10.1080/15267431.2021.1927040 | Qualitative Study  Cross-Sectional | Coping strategies to cope with stress related to the pandemic. | The sample included people on a romantic relationship and cohabiting with their partner. | April 2020 |
| Karagöz et al. (2020) | English | Yes.  https://doi.org/10.1038/s41443-020-00378-4 | Quantitative Study  Cross-sectional | Sexuality in couples during the pandemic. | The sample included married and cohabitating couples. | May 2020 |
| Kolo et al. (2021) | English | Yes.  https://doi.org/10.4236/jss.2021.910001 | Qualitative  Cross-Sectional | Work-family experiences in couples. | The sample included married couples. | During 2020 confinement. |
| Lee et al. (2021) | English | Yes.  <https://doi.org/10.1177/08862605211006359> | Quantitative Study  Longitudinal | Changes in relationship conflict during covid-19 pandemic. | Participants were in an intimate relationship. | March and April 2020 |
| Li & Samp (2021) | English | Yes. https://10.1177/02654075211006199 | Quantitative Study  Cross-Sectional | Conflict and relationship quality during covid-19 pandemic. | The sample included couples living together. | April 1st and May 1st, 2020 |
| Mousavi (2020) | English | Yes.  https://10.3389/fpsyg.2020.553880 | Mixed-Method Study  Cross-Sectional | Psychological well-being, marital  satisfaction, and parental burnout during covid-19 pandemic. | The sample included married parents. | February to  mid-April 2020 |
| Mutang et al. (2022) | English | Yes.  https://doi.org/10.3390/ijerph191811258 | Quantitative Study  Cross Sectional | Stress, and relationship quality during covid-19 pandemic. | The sample included married couples. | September and December 2020 |
| Neff et al. (2021) | English | Yes.  https://10.1177/19485506211022813 | Quantitative Study  Longitudinal | Stress and  relationship quality during covid-19 pandemic. | The sample were composed by married or dating couples. | W1=16th April to 21st May 2020  W2=17th November to 20th December |
| Nuru & Bruess (2021) | English | Yes.  https://10.1177/02654075211037742 | Qualitative Study  Cross-Sectional | Relational coping during struggle during the Covid-19 pandemic. | The sample included married and cohabitating couples. | September 2020 to January, 2021 |
| Omar et al. (2021) | English | Yes  https://doi.org/10.1016/j.esxm.202 100295 | Quantitative Study  Cross-Sectional | Psychological and sexual health during the Covid-19 pandemic. | All participants were married and living together. | 30th March to 30th June, 2020 |
| Osur et al. (2021) | English | Yes  https://doi.org/10.1016/j.esxm.2021.100354 | Quantitative Study  Cross-Sectional | Sexual satisfaction during the Covid-19 pandemic. | All participants were married and living together. | 15th to 30th  September, 2020 |
| Özlü et al. (2021) | English | Yes.  https://doi.org/10.1080/01926187.2021.1941418 | Quantitative Study  Cross-Sectional | Quality of sexual life during the Covid-19 pandemic. | The individuals in this study were married and lived with their partner. | May and June 2020 |
| Panzeri et al. (2020) | English | Yes.  https://10.3389/fpsyg.2020.565823 | Quantitative Study  Cross-Sectional | Changes in sexuality during the Covid-19 pandemic. | The individuals in this study were living with  a partner during the lockdown. | 11th April to 5th May 2020 |
| Partington et al. (2022) | English | Yes.  https://doi.org/10.3389/fpsyg.2022.879195 | Quantitative Study  Cross Sectional | Family functioning, child adjustment, parent-child relationship quality and children’s emotional well-being during the Covid-19 pandemic. | In this study, all parents in the sample were living with their partner in their household. | September and October 2020 |
| Quezada Berumen et al. (2020) | English | Yes https://doi.org/10.24016/2020.v6n3.173 | Quantitative Study  Cross-Sectional | Marital satisfaction during the Covid-19 pandemic. | All couples were living together. | 18th May to 25th May, 2020 |
| Rodríguez-Domínguez (2021) | English | Yes.  <https://doi.org/10.1037/tra0001094> | Quantitative Study  Cross-Sectional | Anxiety, adjustment and relational quality during the Covid-19 pandemic. | All couples were living together. | April 14th to 29th, 2020 |
| Schmid et al. (2021) | English | Yes.  https://doi.org/10.1080/14616696.2020.1836385 | Quantitative Study  Cross-Sectional | Employment and relationship  satisfaction in times of the Covid-19 pandemic | All couples were living together. | Mid-May through early July 2020. |
| Sels et al. (2022) | English | Yes.  https://doi.org/10.5334/ pb.1088 | Cross sectional  Quantitative | Intimate relationships and well-being during the Covid-19 pandemic. | All couples were living together. | From May to August 2020 |
| Seok et al. (2021) | English | Yes.  https://doi.org[10.1177/10105395211014322](https://doi.org/10.1177/10105395211014322) | Quantitative Study  Cross-Sectional | Psychological distress, well-being and relationship quality during the Covid-19 pandemic. | The participants were married or cohabiting. | During the 2020 lockdown. |
| Shockley et al. (2021) | English | Yes. <https://doi.org/10.1037/apl0000857> | Quantitative Study  Longitudinal | Work-Family Strategies during the Covid-19 pandemic. | The sample were composed by married couples, living together. | T1= 18th – 23rd March 2020  T2= 7th – 18th May 2020 |
| Soares et al. (2021) | English | Yes. <https://doi.org/10.1089/jwh.2020.8903> | Quantitative Study  Cross-Sectional | Dual-Physician Couples during the Covid-19 pandemic. | Respondents were members of a dual-physician couples, living together. | 30th April – 26th May |
| James et al. (2022) | English | Yes. https://doi.org/10.1111/fare.12705 | Quantitative Study  Cross sectional | Relationship satisfaction during the Covid-19 pandemic. | Respondents were married  and cohabiting. | September and October of 2020 |
| Tan (2021) | English | Yes.  https://doi.org/10.1016/j.jsxm.2021.12.004 | Quantitative Study  Longitudinal | Marital sexual activity during the Covid-19 pandemic. | The sample were composed by married women living with their partner. | May 2020 (T1) June 2020 (T2) |
| Tomohiro (2021) | English | Yes.  <https://doi.org/10.4337/9781803929507.00011> | Quantitative Study  Longitudinal | Work-family balance during the Covid-19 pandemic. | The sample were married. | April, May, August, and December 2020 |
| Turliuc & Candel (2021) | English | Yes.  https://doi.org/10.3389/fpsyg.2021.635148 | Quantitative Study  Longitudinal | Marital stress and satisfaction during the Covid-19 pandemic. | The sample included 144 married couples. | T1 – 16th March,2020  T2 – 15th May, 2020 |
| Vowels et al. (2021) | English | Yes.  https://doi.org/10.1177/02654075211041412 | Mixed-Method Study  Longitudinal | Change of romantic relationships during the Covid-19 pandemic. | The sample consisted of married and cohabitating couples. | March 30th, 2020, and April 21st, 2020, |
| Waddell et al. (2021) | English | Yes. https://doi.org/10.1177/0265407521996476 | Quantitative Study  Longitudinal | Division of Labor at Home during the Covid-19 pandemic. | This study included 234 dyads in long term relationships. | 8th to 27th April, 2020 |
| Weber et al. (2020) | English | Yes.  https://doi.org/10.1111/famp.12700 | Quantitative Study  Cross-Sectional | Individual and relational outcomes during the Covid-19 pandemic. | All couples were living together. | 2nd May to 11th May, 2020 |
| Williamson (2020) | English | Yes.  https://doi.org/10.1177/0956797620972688 | Quantitative Study  Longitudinal | Relationship satisfaction during the Covid-19 pandemic. | The sample were composed by people in a relationship. 91% of the couples were living together. | December 2019, March, and April 2020 |
| Wong et al. (2022) | English | Yes.  https://doi.org/10.1093/geronb/gbac140 | Longitudinal  Quantitative | Relationship quality during the Covid-19 pandemic. | The sample were constituted by respondents  married or living with a partner. | September 2020 and January 2021 |
| Zamarro & Prados (2021) | English | Yes.  https://doi.org/10.1007/s11150-020-09534-7 | Quantitative Study  Longitudinal | Division of household tasks and well-being during the Covid-19 pandemic. | The sample were composed by respondents who reported being married or living together with their partners in the same household. | 10th March to 22nd July, 2020 |
| Zhang et al. (2021) | English | Yes.  https://doi.org/10.1016/j.jsxm.2021.06.013 | Quantitative Study  Cross-Sectional | Mental health, stress and sexuality in couples during the Covid-19 pandemic. | The sample were compiled by couples living together during the pandemic. | March, 2020 |

**Table 13. Inclusion Criteria for Studies Targeting Parents**

| **Date of Publication** | **Language** | **Published in Journal with Peer Review** | **Empirical Study** | **Target of the Study** | **Sample Characteristics** | **Data Collection** |
| --- | --- | --- | --- | --- | --- | --- |
| Carlson et al. (2020) | English | Yes. https://[10.1111/soin.12459](https://doi.org/10.1111/soin.12459) | Quantitative Study  Cross-Sectional | Domestic Labor During the Early Days during the Covid-19 pandemic. | Parents with children with a mean age of the 9.63 years old. | April 2020 |
| Carvalho & Matias (2023) | English | Yes. https://doi.org/10.1007/s12144-023-04658-2 | Quantitative Study  Cross Sectional | Parental exhaustion, relationship outcomes and dyadic coping during the Covid-19 pandemic. | Parents with children less than a year to 17 years old. | April to June 2020 |
| Chung et al. (2020) | English | Yes. https://doi.org/10.1007/s10896-020-00200-1 | Quantitative Study  Cross Sectional | Parents’ perceived impact of  COVID-19 on parent-child relationships and the effect of parenting stress during the Covid-19 pandemic. | Parents with children at most 12 years old. | 22nd April to  5th, 2020 |
| Feinberg et al. (2022) | English | Yes.  <https://doi.org/10.1111/famp.12649> | Quantitative Study Longitudinal | Parent, Child, and Family Functioning during the Covid-19 pandemic. | Parents with children on average 9.9 years old. | April and May 2020 |
| Hanetz-Gamliel et al. | English | Yes. https://doi.org/10.1007/s10826-021-02115-x | Quantitative Study  Cross-Sectional | Anxiety and parenting during the Covid-19 pandemic. | Parents with children between the ages of 3 and 12 years old. | Mid-March until the end of April 2020 |
| Hiraoka & Tomoda (2020) | English | Yes.  <https://doi.org/10.1111/pcn.13088> | Quantitative Study  Cross-Sectional | Parenting stress during the Covid-19 pandemic. | Parents with children between 6 and 8 years old. | 29th to 30th April |
| Hood et al. (2021) | English | Yes.  https://doi.org/10.3390/ijerph182312865 | Qualitative study  Cross-Sectional | Family routines and parent relationships during the Covid-19 pandemic. | Parents with children were aged 3 to 9 years old. | End of July 2020 |
| Idsøe et al. (2021) | English | Yes. https://doi.org/[10.3389/fpsyt.2021.701782](https://doi.org/10.3389/fpsyt.2021.701782) | Quantitative Study  Longitudinal | Pandemic stress and parenting during the Covid-19 pandemic. | Parents with adolescents were 11 to 13 years old. | Spring, 2020 |
| McRae (2021) | English | Yes.  <https://doi.org/10.1037/dev0001207> | Quantitative Study  Cross-Sectional | Distress, partner support and coparenting during the Covid-19 pandemic. | The parents had children from 4 to 5 years old. | 26th March to 28th April, 2020 |
| Partington et al. (2022) | English | Yes.  https://doi.org/10.3389/fpsyg.2022.879195 | Quantitative Study  Cross Sectional | Family functioning, child adjustment, parent-child relationship quality and children’s emotional well-being during the Covid-19 pandemic. | The parents had children between the ages of 2–18 years old. | September and October 2020 |
| Spinelli et al. (2020) | English | Yes. https://doi.org/10.1111/famp.12601 | Quantitative Study  Longitudinal | Parenting stress during the covid-19 pandemic during the Covid-19 pandemic. | Parents with children aged between 2 and 14 years old. | 2nd – 7th April, 2020 |
| Zamarro & Prados (2021) | English | Yes.  https://doi.org/10.1007/s11150-020-09534-7 | Quantitative Study  Longitudinal | Division of household tasks and well-being during the Covid-19 pandemic. | About 47% of respondents in the sample reported having school-age children. | 10th March to 22nd July, 2020 |
