# Supplementary material for: Couples and parenting dynamics during Covid-19 pandemic: A systematic review of the literature
Source: PLoS One. 2025 Feb 18;20(2):e0315417. doi: 10.1371/journal.pone.0315417 (PMC11835339; doi:10.1371/journal.pone.0315417)
Supplement: S6 Table — Table 14. Excluded reports for the systematic review of the literature. (DOCX) [file pone.0315417.s008.docx]

**Table 14. Excluded Reports for the Systematic Review of the Literature**

| **Name of the study** | **Reason to exclude** | **Doi** |
| --- | --- | --- |
| 1. The Global Impact of the COVID-19 Pandemic on Individuals' and Couples' Sexuality | Study Characteristics | https://doi.org/[10.3389/fpsyg.2021.798260](https://doi.org/10.3389/fpsyg.2021.798260) |
| 1. Family Science in the Context of the COVID-19 Pandemic: Solutions and New Directions | Study Characteristics | <https://doi.org/10.1111/famp.12582> |
| 1. Implications of social isolation, separation, and loss during the COVID-19 pandemic for couples’ relationships | Study Characteristics | https://doi.org/[10.1016/j.copsyc.2021.07.014](https://doi.org/10.1016/j.copsyc.2021.07.014) |
| 1. Not in a romantic relationship: Love in Quarantine: Sexting, Stress, and Coping During the COVID‑19 Lockdown | Sample characteristics | https://doi.org/10.1007/s13178-021-00645-z |
| 1. Stress and parenting during the global COVID-19 pandemic. | Sample characteristics | https://doi.org/[10.1016/j.chiabu.2020.104699](https://doi.org/10.1016/j.chiabu.2020.104699) |
| 1. COVID-19-Related Conflict in Couples with Young Children. | Sample characteristics | <https://doi.org/10.31235/osf.io/cpkj6> |
| 1. Parenting under pressure: A mixed-methods investigation of the impact of COVID-19 on family life | Sample characteristics | https://doi.org/10.1016/j.jadr.2021.100161 |
| 1. Relationship status, single parents: Stress and parenting during the global COVID-19 pandemic | Sample characteristics | <https://doi.org/10.1016/j.chiabu.2020.104699> |
| 1. Substance use disorder treatment, parenting, and COVID-19 | Not an empirical study. | https://doi.org/10.1016/j.jsat.2020.108148 |
| 1. The Impact of the COVID-19 Quarantine on Sexual Life in Italy | Sample characteristics | https://doi.org/10.1016/j.urology.2020.06.101 |
| 1. Love at the time of the Covid-19 pandemic: preliminary results of an online survey conducted during the quarantine in Italy | Sample characteristics | https://doi.org/10.1038/s41443-020-0305-x |
| 1. COVID-19 and the Well-being of Children and Families | Study Characteristics | https://doi.org/10.1542/peds.2020-022079 |
| 1. Women’s Sexual Health During the Pandemic of COVID-19: Declines in Sexual Function and Sexual Pleasure | Study Characteristics | https://doi.org/10.1007/s11930-021-00309-4 |
| 1. From “It Has Stopped Our Lives” to “Spending More Time Together Has Strengthened Bonds”: The Varied Experiences of Australian Families During COVID-19. | Sample characteristics | https://doi.org/10.3389/fpsyg.2020.588667 |
| 1. Reaching Up, Down, In, and Around: Couple and Family Coping During the Coronavirus Pandemic | Sample characteristics | https://doi.org/10.1111/famp.12570 |
| 1. COVID-19 and Parent-Child Psychological Well-being | Sample characteristics | <https://doi.org/10.1542/peds.2020-007294> |
| 1. Parenting in a Pandemic: Work–Family Arrangements, Well‐Being, and Intimate Relationships Among Adoptive Parents | Sample characteristics | https://doi.org/10.1111/fare.12528 |
| 1. A Mixed‐method Study of Individual, Couple, and Parental Functioning During the State‐regulated COVID‐19 Lockdown in Spain | Sample characteristics | https://doi.org/10.1111/famp.12585 |
| 1. Parenthood as a driver of increased gender inequality during COVID-19? Exploratory evidence from Germany | Sample characteristics | https://doi.org/10.1080/14616696.2020.1833229 |
| 1. Impact of the COVID-19 pandemic on the sexual behavior of the population | Study characteristics | https://doi.org/10.1590/s1677-5538.ibju.2020.s116 |
| 1. Does the COVID-19 pandemic impact parents’ and adolescents’ well-being? An EMA-study on daily affect and parenting. | Sample characteristics | https://doi.org/10.1371/journal.pone.0240962 |
| 1. Parenting in a Pandemic: Parental Stress During the Physical Distancing Intervention Following the onset of the COVID-19 Outbreak | Sample characteristics | https://doi.org/10.31234/osf.io/3nsda |
| 1. COVID-19 pandemic and the quality of couples’ sexual relationships | Sample characteristics | <https://doi.org/10.38053/acmj.848051> |
| 1. A Study of Parents’ Experiences Across Gender and Income Levels | Sample characteristics | https://doi.org/10.1111/fare.12571 |
| 1. Changes in Sex Life among People in Taiwan during the COVID-19 Pandemic: The Roles of Risk Perception, General Anxiety, and Demographic Characteristics | Sample characteristics | https://doi.org/10.3390/ijerph17165822 |
| 1. Infidelity in the Time of COVID‐19. | Study characteristics | <https://doi.org/10.1111/famp.12576> |
| 1. Les impacts du confinement lié au coronavirus sur la sexualité | Language characteristics | https://doi.org/10.1016/j.sexol.2020.08.002 |
| 1. Family in the Age of COVID‐19 | Study characteristics | https://doi.org/10.1111/famp.12543 |
| 1. Less Sex, but More Sexual Diversity: Changes in Sexual Behavior during the COVID-19 Coronavirus Pandemic | Study characteristics | https://doi.org/10.1080/01490400.2020.1774016 |
| 1. Challenges in the Practice of Sexual Medicine in the Time of COVID-19 in China | Sample characteristics | https://doi.org/10.1016/j.jsxm.2020.04.380 |
| 1. Jealousy and Electronic Intrusion Mediated by Relationship Uncertainty in Married and Cohabiting Couples During COVID-19 | No access | https://doi.org/10.1089/cyber.2020.0669 |
| 1. COVID-19 and Sexuality: Reinventing Intimacy | Study characteristics | https://doi.org/10.1007/s10508-020-01796-7 |
| 1. Romantic Relationship Conflict Due to the COVID-19 Pandemic and Changes in Intimate and Sexual Behaviors in a Nationally Representative Sample of American Adults | Sample characteristics | https://doi.org/10.1080/0092623x.2020.1810185 |
| 1. Parenting-Related Exhaustion During the Italian COVID-19 Lockdown | Sample characteristics | https://doi.org/10.1093/jpepsy/jsaa093 |
| 1. Early impacts of the COVID-19 pandemic on sexual behaviour in Britain: findings from a large, quasi-representative survey (Natsal-COVID). | Sample characteristics | https://doi.org/10.1136/sextrans-2021-sti.78 |
| 1. Desire for parenthood at the time of COVID-19 pandemic: an insight into the Italian situation | Sample characteristics | https://doi.org/10.1080/0167482x.2020.1759545 |
| 1. Parents and Children During the COVID-19 Lockdown: The Influence of Parenting Distress and Parenting Self-Efficacy on Children’s Emotional Well-Being | Sample characteristics | https://doi.org/10.3389/fpsyg.2020.584645 |
| 1. A Little Autonomy Support Goes a Long Way: Daily Autonomy‐Supportive Parenting, Child Well‐Being, Parental Need Fulfillment, and Change in Child, Family, and Parent Adjustment Across the Adaptation to the COVID‐19 Pandemic. | Sample characteristics | https://doi.org/10.1111/cdev.13515 |
| 1. Impact of COVID-19 on adolescents’ mental health: a systematic review. | Study Characteristics | https://doi.org/10.1186/s43045-020-00075-4 |
| 1. Relationship Between Parenting Practices and Children’s Screen Time During the COVID-19 Pandemic in Turkey | Sample characteristics | <https://doi.org/10.1016/j.pedn.2020.10.00> |
| 1. Relationship quality and mental health during COVID-19 lockdown. | Sample characteristics | https://doi.org/10.1371/journal.pone.0238906 |
| 1. Risk and resilience in family well-being during the COVID-19 pandemic. | Study Characteristics | https://doi.org/10.1037/amp0000660 |
| 1. Love in the Time of Corona: Heterosexual Romance, Space, and Society in Japanese Fiction on COVID-19 | Study Characteristics | https://doi.org/10.5195/jll.2021.214 |
| 1. COVID‐19 Pandemic: Applying a Multisystemic Lens | Study Characteristics | https://doi.org/10.1111/famp.12584 |
| 1. Supporting families to protect child health: Parenting quality and household needs during the COVID-19 pandemic. | Sample characteristics | https://doi.org/10.1371/journal.pone.0251720 |
| 1. “It Strengthened My Core Relationships and Filtered Out the Rest:” Intimacy Communication During COVID-19. | Sample characteristics | https://doi.org/10.1007/s12119-021-09890-1 |
| 1. The COVID‐19 Pandemic and Families in Japan. | Study Characteristics | https://doi.org/10.1002/anzf.1438 |
| 1. Families in the Time of the Pandemic: Breakdown or Breakthrough? | Study Characteristics | https://doi.org/10.1002/anzf.1445 |
| 1. The Impact of Social Distancing Measures Due to COVID-19 Pandemic on Sexual Function and Relationship Quality of Couples in Greece | Sample characteristics | https://doi.org/10.1016/j.esxm.2021.100364 |
| 1. Thematic Analysis of Parent–Child Conversations About COVID-19: “Playing It Safe.” | Sample characteristics | https://doi.org/10.1007/s10826-020-01889-w |
| 1. Helping Couples Connect during the COVID‐19 Pandemic: A Pilot Randomised Controlled Trial of an Awareness, Courage, and Love Intervention | Study Target | <https://doi.org/10.1111/aphw.12241> |
| 1. Parent–Child Relationships and the COVID-19 Pandemic: An Exploratory Qualitative Study with Parents in Early, Middle, and Late Adulthood | Sample characteristics | https://doi.org/10.1007/s10804-021-09381-5 |
| 1. Parenting and child and adolescent mental health during the COVID-19 pandemic. | Sample characteristics | <https://doi.org/10.31234/osf.io/ag2r7> |
| 1. Mental health status of students’ parents during COVID-19 pandemic and its influence factors | Sample characteristics | <https://doi.org/10.1136/gpsych-2020-100250> |
| 1. Surviving Marital Relationship During the COVID-19 Pandemic: A Systematic Review on Marital Conflict | Study Characteristics | https://doi.org/[10.2991/assehr.k.210423.015](https://doi.org/10.2991/assehr.k.210423.015) |
| 1. Turning back the clock: Beliefs about gender roles during lockdown | Study Characteristics | https://doi.org/10.1016/j.labeco.2023.102363 |
| 1. Relationship satisfaction in the early stages of the COVID-19 pandemic: Cross-national examination of situational, dispositional, and relationship factors | Sample characteristics | https://doi.org/10.1371/journal.pone.0264511 |
| 1. Couples Satisfaction during the Covid-19 Pandemic: a Systematic Review | Study Characteristics | www.psychologyandeducation.net |
| 1. Impact of COVID-19 Related Stress on Sexual Desire and Behavior in a Canadian Sample | Study Characteristics | https://doi.org/10.1080/19317611.2021.1947932 |
| 1. A descriptive literature review of early research on COVID-19 and close relationships | Study Characteristics | https://doi.org/10.1177/02654075221115387 |
| 1. COVID-19 and Romantic Relationships | Study Characteristics | https://doi.org/10.3390/encyclopedia1040079 |
| 1. Love in the time of COVID-19:A systematic mapping review of empirical research on romantic relationships one year into the COVID-19 pandemic | Study Characteristics | https://doi.org/10.1111/famp.12775 |
| 1. Locked-Down Love: A Study of Intimate Relationships Before and After the COVID Lockdown | Sample characteristics | https://doi.org/10.1111/fare.12582 |
| 1. An Investigation of Sexual and Relationship Adjustment During COVID‑19 | Sample characteristics | https://doi.org/10.1007/s10508-021-02212-4 |
| 1. Parenting during the COVID-19 Lockdown in Portugal: 2. Changes in Daily Routines, Co-Parenting Relationships, Emotional Experiences, and Support Networks | Sample characteristics | https://doi.org/10.3390/children8121124 |
| 1. Parenting Adolescents in Times of a Pandemic: Changes in Relationship Quality, Autonomy Support, and Parental Control? | Sample characteristics | https://doi.org/10.1037/dev0001208 |
| 1. Experiences With COVID-19 Stressors and Parents’ Use of Neglectful, Harsh, and Positive Parenting Practices in the Northeastern United States | Sample characteristics | https://doi.org/10.1177/10775595211006465 |
| 1. Parenting During the COVID‑19 Pandemic in Portugal: The Mediating Role of Work‑Family Guilt in the Relationship Between Self‑Compassion and Mindful Parenting in a Sample of Working Mothers | Sample characteristics | https://doi.org/10.1007/s12671-023-02114-7 |
| 1. Parenting stress, self-efficacy and COVID-19 health risks as predictors of general stress among nurses | Sample characteristics | https://doi.org/10.1111/ijn.13009 |
| 1. Parental stress, food parenting practices and child snack intake during the COVID-19 pandemic | Sample characteristics | https://doi.org/10.1016/j.appet.2021.105119 |
| 1. Parenting During the COVID-19 Pandemic | Study Characteristics | https://doi.org/10.31729/jnma.5319 |
| 1. Parenting and parent–child home practice during the COVID‑19 pandemic: a case in central China | Sample characteristics | https://doi.org/10.1038/s41598-023-45726-8 |
| 1. Well-being of Parents and Children During the COVID-19 Pandemic: A National Survey | Sample characteristics | https://doi.org/[10.1542/peds.2020-016824](https://doi.org/10.1542/peds.2020-016824) |
| 1. Parenting and Children’s Behavior During the COVID 19 Pandemic: Mother’s Perspective | Sample characteristics | https://doi.org/10.3389/fpsyg.2022.801614 |
| 1. Parenting, mental health, and Covid-19: A rapid systematic review | Study Characteristics | https://doi.org/[10.1542/peds.2020-016824](https://doi.org/10.1542/peds.2020-016824) |
| 1. Parental Challenges During the COVID‑19 Pandemic: Psychological Outcomes and Risk and Protective Factors | Study Characteristics | https://doi.org/10.1007/s11920-023-01412-0 |
| 1. Abusive and positive parenting behavior in Japan during the COVID-19 pandemic under the state of emergency | Sample characteristics | https://doi.org/10.1016/j.chiabu.2021.105212 |
| 1. Parenting practices, stressors and parental concerns during COVID-19 in Pakistan | Sample characteristics | https://doi.org/10.1016/j.chiabu.2021.105393 |
| 1. Couples and COVID-19 vaccination: Frequency and reasons for discordance | Target of the Study | <https://doi.org/10.1016/j.vaccine.2022.02.055> |
| 1. Love and Infidelity: Causes and Consequences | Study characteristics | <https://doi.org/10.3390/ijerph20053904> |
| 1. COVID-19 suicidal behavior among couples and suicide pacts: Case study evidence from press reports | Study characteristics | <https://doi.org/10.1016/j.psychres.2020.113105> |
| 1. Depression, anxiety, and stress in infertile couples during the COVID-19 pandemic: the consequences we face | Sample characteristics | https://doi.org/10.5935/1518-0557.20230018 |
| 1. Divorced and separated parents during the COVID-19 pandemic | Sample characteristics | https://doi.org/[10.1111/famp.12693](https://doi.org/10.1111/famp.12693) |
| 1. Intermarriage and COVID-19 mortality among immigrants. A population-based cohort study from Sweden | Target of Study | https://doi.org/10.1136/bmjopen-2021-048952 |
| 1. Relationship difficulties and “technoference” during the COVID-19 pandemic | Sample characteristics | <https://doi.org/10.1177/026540752210936> |
| 1. Helping Couples Connect during the COVID-19 Pandemic: A Pilot Randomised Controlled Trial of an Awareness, Courage, and Love Intervention | Target of Study | https://doi.org/10.1111/aphw.1224 |
| 1. The work-family interface and the COVID-19 pandemic: A systematic review | Study characteristics | https://doi.org/[10.3389/fpsyg.2022.914474](https://doi.org/10.3389/fpsyg.2022.914474) |
| 1. Risk and resilience in couple’s adjustment to the COVID-19 pandemic | Sample characteristics | https://doi.org/10.1177/02654075221094556 |
| 1. The immediate impact of lockdown measures on mental health and couples’ relationships during the COVID-19 pandemic - results of a representative population survey in Germany | Sample characteristics | <https://doi.org/10.1016/j.socscimed.2021.113954> |
| 1. Working from Home and COVID-19: The Chances and Risks for Gender Gaps | Study characteristics  Sample characteristics | https://doi.org/10.1007/s10272-020-0938-5 |
| 1. Helping Couples in the Shadow of COVID-19 | Study characteristics | https://doi.org/10.1111/famp.12575 |
| 1. Changes in marriage, divorce and births during the COVID-19 pandemic in Japan | Study characteristics Target of the Study | https://doi.org/10.1136/bmjgh-2021-007866 |
| 1. Clinical Supervision of Couple and Family Therapy during COVID-19 | Study characteristics | https://doi.org/[10.1111/famp.12591](https://doi.org/10.1111/famp.12591) |
| 1. The Effects of the COVID-19 Pandemic on the Parenting of Infants: A Couples Study | Sample Characteristics | <https://doi.org/10.3390/ijerph192416883> |
| 1. Sexuality, sexual well being, and intimacy during COVID-19 pandemic: An advocacy perspective | Study characteristics | https://doi: [10.4103/psychiatry.IndianJPsychiatry_484_20](https://doi.org/10.4103/psychiatry.IndianJPsychiatry_484_20) |
| 1. Eating behaviors and body self-esteem in couple’s satisfaction during COVID-19 quarantine | Sample Characteristics | https://doi.org/10.1177/02601060231166162 |
| 1. Attachment-based family therapy in the age of telehealth and COVID-19 | Target of the Study | https://doi.org/[10.1111/jmft.12509](https://doi.org/10.1111/jmft.12509) |
| 1. Couples Therapists' Attitudes Toward Online Therapy During the COVID-19 Crisis | Target of the Study | https://doi.org/[10.1111/famp.12647](https://doi.org/10.1111/famp.12647) |
| 1. Couple and family therapists' experiences with Telehealth during the COVID-19 pandemic: a phenomenological analysis | Target of the Study | https://doi.org/[10.1007/s10591-022-09640-x](https://doi.org/10.1007/s10591-022-09640-x) |
| 1. The impact of the COVID-19 pandemic on perinatal loss among Italian couples: A mixed-method study | Sample Characteristics | https://doi.org/[10.3389/fpsyg.2022.929350](https://doi.org/10.3389/fpsyg.2022.929350) |
| 1. Anxiety, past trauma and changes in relationships in Japan during COVID-19 | Sample Characteristics | <https://doi.org/10.1016/j.jpsychires.2022.04.032> |
| 1. Physician Burnout in Primary Care during the COVID-19 Pandemic: A Cross-Sectional Study in Portugal | Sample Characteristics | <https://doi.org/10.1177/215013272110084> |
| 1. Teleworking in Portuguese communities during the COVID-19 pandemic | Sample Characteristics | https://doi.org/10.1108/JEC-06-2020-0113 |
| 1. Parental Burnout and the COVID-19 Pandemic: How Portuguese Parents Experienced Lockdown Measures | Sample Characteristics | https://doi.org/10.1111/fare.12558 |
| 1. Changes in sexual activities, function, and satisfaction during the COVID-19 pandemic era: a systematic review and meta-analysis | Study Characteristics | https://doi.org/10.1093/sexmed/qfad005 |
| 1. The COVID‑19 pandemic and family business performance | Target of the Study | https://doi.org/[10.1007/s11187-023-00766-2](https://doi.org/10.1007/s11187-023-00766-2) |
| 1. The COVID-19 Pandemic: A Family Affair | Study characteristics | https://doi.org/[10.1177/1074840720920883](https://doi.org/10.1177/1074840720920883) |
| 1. Stroke Family Caregiving and the COVID-19 Pandemic: Impact and Future Directions | Study characteristics | <https://doi.org/10.1161/120.033525> |
| 1. COVID-19 Pandemic Practices, Payment Models, and Publication Successes: Family Medicine Studies a Variety of Primary Care Questions | Study characteristics  Target of the Study | https://doi.org/10.3122/jabfm.2021.03.210099. |
| 1. Family physician leadership during the COVID-19 pandemic: roles, functions and key supports | Target of the Study Sample Characteristics | https://doi.org/10.1108/LHS-03-2022-0030 |
| 1. Impact of the COVID-19 Pandemic on Patient- and Family-Centered Care and on the Mental Health of Health Care Workers, Patients, and Families | Study Target | <https://doi.org/10.3389/fped.2022.880686> |
| 1. Family Caregiving During the COVID-19 Pandemic | Sample Characteristics | https://doi.org/[10.1093/geront/gnab049](https://doi.org/10.1093/geront/gnab049) |
| 1. Family economic hardship and adolescent mental health during the COVID-19 pandemic | Target of the Study | <https://doi.org/10.3389/fpubh.2022.904985> |
| 1. COVID-19 pandemic stresses and relationships in college students | Sample Characteristics | https://doi.org/10.1111/fare.12602 |
| 1. Family Resilience during COVID-19 Pandemic: A Literature Review | Study characteristics | https://doi.org/[10.1177/10664807211023875](https://doi.org/10.1177/10664807211023875) |
| 1. Family member incarceration and coping strategies during the COVID-19 pandemic | Sample Characteristics | https://doi.org/10.1186/s40352-021-00142-w |
| 1. COVID-19 pandemic impact on family life and exacerbated emotional and behavioral health among preschool children: A longitudinal study | Sample Characteristics | https://doi.org/[10.1016/j.ijchp.2022.100327](https://doi.org/10.1016/j.ijchp.2022.100327) |
| 1. Family Well-Being During the COVID-19 Pandemic: The Risks of Financial Insecurity and Coping | Sample Characteristics | https://doi.org/10.1111/jora.12776 |
| 1. Family Structure and Adolescent Mental Health Service Utilization During the COVID-19 Pandemic | Target of the Study | https://doi.org/[10.1016/j.jadohealth.2023.01.018](https://doi.org/10.1016/j.jadohealth.2023.01.018) |
| 1. Family ownership during the Covid-19 pandemic | Target of the Study  Sample characteristics | https://doi.org/[10.1016/j.jbankfin.2021.106385](https://doi.org/10.1016/j.jbankfin.2021.106385) |
| 1. Prevalence and factors associated with family planning during COVID-19 pandemic in Bangladesh: A cross-sectional study | Target of the Study  Sample characteristics | https://doi.org/[10.1371/journal.pone.0257634](https://doi.org/10.1371/journal.pone.0257634) |
| 1. Family caregiving during the COVID-19 pandemic: factors associated with anxiety and depression of carers for community dwelling older adults in Hong Kong | Target of the Study | https://doi.org/[10.1186/s12877-021-02741-6](https://doi.org/10.1186/s12877-021-02741-6) |
| 1. Pandemic Precarity: COVID-19’s Impact on Mexican and Central American Immigrant Families | Sample characteristics | https://doi.org/10.1111/jomf.12930 |
| 1. Disrupted Family Plans and Exacerbated Inequalities Associated With COVID-19 Pandemic | Study characteristics | https://doi.org/[10.1001/jamanetworkopen.2021.24399](https://doi.org/10.1001/jamanetworkopen.2021.24399) |
| 1. Family Communication and Psychological Distress in the Era of COVID-19 Pandemic: Mediating Role of Coping | Sample characteristics | https://doi.org/[10.1177/0192513X211044489](https://doi.org/10.1177/0192513x211044489) |
| 1. Differences in family functioning before and during the COVID-19 pandemic: an observational study in Peruvian families | Target of the Study  Sample characteristics | https://doi.org/[10.7717/peerj.16269](https://doi.org/10.7717/peerj.16269) |
| 1. Changes in Family Chaos and Family Relationships during the COVID-19 Pandemic: Evidence from a Longitudinal Study | Sample characteristics | https://doi.org/[10.1037/dev0001217](https://doi.org/10.1037/dev0001217) |
| 1. Work-family and family-work conflict and stress in times of COVID-19 | Sample characteristics | https://doi.org/[10.3389/fpsyg.2022.951149](https://doi.org/10.3389/fpsyg.2022.951149) |
| 1. Family relationship of nurses in COVID-19 pandemic: A qualitative study | Target of the Study  Sample characteristics | https://doi.org/[10.12998/wjcc.v10.i19.6472](https://doi.org/10.12998/wjcc.v10.i19.6472) |
| 1. Disordered gaming, loneliness, and family harmony in gamers before and during the COVID-19 pandemic | Sample characteristics | https://doi.org/[10.1016/j.abrep.2022.100426](https://doi.org/10.1016/j.abrep.2022.100426) |
| 1. The relationship between family variables and family social problems during the COVID-19 pandemic | Sample characteristics | https://doi.org/[10.1371/journal.pone.0270210](https://doi.org/10.1371/journal.pone.0270210) |
| 1. Family Resilience and Adolescent Mental Health during COVID-19: A Moderated Mediation Model | Target of the Study | https://doi.org/[10.3390/ijerph19084801](https://doi.org/10.3390/ijerph19084801) |
| 1. The changes in family functioning and family happiness during the COVID-19 pandemic: The situation in Thailand | Sample characteristics | https://doi.org/[10.3389/fpubh.2022.1055819](https://doi.org/10.3389/fpubh.2022.1055819) |
| 1. Life Interrupted: Family Routines Buffer Stress during the COVID-19 Pandemic | Sample characteristics | https://doi.org/[10.1007/s10826-021-02063-6](https://doi.org/10.1007/s10826-021-02063-6) |
| 1. The Effects of Depression and Fear in Dual-Income Parents on Work-Family Conflict During the COVID-19 Pandemic | Sample characteristics | https://doi.org/[10.1177/21582440231157662](https://doi.org/10.1177/21582440231157662) |
| 1. Enduring COVID-19 lockdowns: Risk versus resilience in parents’ health and family functioning across the pandemic | Sample characteristics | https://doi.org/[10.1177/02654075221095781](https://doi.org/10.1177/02654075221095781) |
| 1. Family relationship quality during the COVID-19 pandemic: The value of adolescent perceptions of change | Target of the Study  Sample characteristics | https://doi.org/[10.1016/j.adolescence.2021.11.005](https://doi.org/10.1016/j.adolescence.2021.11.005) |
| 1. Teacher Teleworking during the COVID-19 Pandemic: Association between Work Hours, Work–Family Balance and Quality of Life | Sample characteristics | https://doi.org/[10.3390/ijerph18147566](https://doi.org/10.3390/ijerph18147566) |
| 1. The Association of COVID-19 Stressors and Family Health on Overeating before and during the COVID-19 Pandemic | Sample characteristics | https://doi.org/[10.3390/ijerph19106174](https://doi.org/10.3390/ijerph19106174) |
| 1. Association Between Time Spent With Family and Loneliness Among Japanese Workers During the COVID-19 Pandemic: A Cross-Sectional Study | Sample characteristics | https://doi.org/[10.3389/fpsyt.2021.786400](https://doi.org/10.3389/fpsyt.2021.786400) |
| 1. Implications of the COVID-19 Pandemic on the Family Structural Dimensions: A Correlational Study | Sample characteristics | https://doi.org/[10.3390/ejihpe13090115](https://doi.org/10.3390/ejihpe13090115) |
| 1. Experiences of Work-Family Conflict and Mental Health Symptoms by Gender Among Physician Parents During the COVID-19 Pandemic | Sample characteristics | https://doi.org/[10.1001/jamanetworkopen.2021.34315](https://doi.org/10.1001/jamanetworkopen.2021.34315) |
| 1. Colliding worlds: Family carers’ experiences of balancing work and care in Ireland during the COVID-19 pandemic | Sample characteristics | https://doi.org/[10.1111/hsc.13365](https://doi.org/10.1111/hsc.13365) |
| 1. Family Cohesion and Sleep Disturbances During COVID‑19: the Mediating Roles of Security and Stress | Sample characteristics | https://doi.org/[10.1007/s11469-022-00753-w](https://doi.org/10.1007/s11469-022-00753-w) |
| 1. The Predictive Effects of Family and Individual Wellbeing on University Students' Online Learning During the COVID-19 Pandemic | Sample characteristics | https://doi.org/[10.3389/fpsyg.2022.898171](https://doi.org/10.3389/fpsyg.2022.898171) |
| 1. Romantic Relationships and Mental Health During the COVID-19 Pandemic in Austria: A Population-Based Cross-Sectional Survey | Sample characteristics | https://doi.org/[10.3389/fpsyg.2022.857329](https://doi.org/10.3389/fpsyg.2022.857329) |
| 1. Young people’s romantic relationships and sexual activity before and during the COVID-19 pandemic | Sample characteristics | https://doi.org/[10.1186/s12889-021-11818-1](https://doi.org/10.1186/s12889-021-11818-1) |
| 1. Perceptions of Relationship Quality Before and During COVID‑19 Pandemic Among Young Sexual Minority Men in Romantic Relationships | Sample characteristics | https://doi.org/[10.1007/s10508-021-02254-8](https://doi.org/10.1007/s10508-021-02254-8) |
| 1. Psychophysical Impact of COVID-19 Pandemic and Same-Sex Couples' Conflict: The Mediating Effect of Internalized Sexual Stigma | Sample characteristics | https://doi.org/[10.3389/fpsyg.2022.860260](https://doi.org/10.3389/fpsyg.2022.860260) |
| 1. Love under lockdown: How changes in time with partner impacted stress and relationship outcomes during the COVID-19 pandemic | Sample characteristics | https://doi.org/[10.1177/02654075231162599](https://doi.org/10.1177/02654075231162599) |
| 1. The ABC-X’s of Stress among U.S. Emerging Adults during the COVID-19 Pandemic: Relationship Quality, Financial Distress, and Mental Health | Sample characteristics | https://doi.org/[10.3390/ijerph192013125](https://doi.org/10.3390/ijerph192013125) |
| 1. Parenting and Adjustment Problems among Preschoolers during COVID-19 | Sample characteristics | https://doi.org/[10.1007/s10826-022-02439-2](https://doi.org/10.1007/s10826-022-02439-2) |
| 1. Family Disruption and Parenting During the COVID-19 Pandemic | Sample characteristics | https://doi.org/[10.1177/0192513X211042852](https://doi.org/10.1177/0192513x211042852) |
| 1. Parenting practices and adolescent delinquency: COVID-19 impact in the United States | Sample characteristics | https://doi.org/[10.1016/j.childyouth.2022.106791](https://doi.org/10.1016/j.childyouth.2022.106791) |
| 1. Parenting practices, stressors and parental concerns during COVID-19 in Pakistan | Sample characteristics | https://doi.org/[10.1016/j.chiabu.2021.105393](https://doi.org/10.1016/j.chiabu.2021.105393) |
| 1. Mothers’ and fathers’ parenting attitudes during COVID-19 | Sample characteristics | https://doi.org/[10.1007/s12144-021-01605-x](https://doi.org/10.1007/s12144-021-01605-x) |
| 1. Maternal distress and parenting during COVID-19: diferential efects related to pre-pandemic distress? | Target of the Study | https://doi.org/[10.1186/s12888-023-04867-w](https://doi.org/10.1186/s12888-023-04867-w) |
| 1. Parenting in a time of COVID-19 | Study characteristics | https://doi.org/[10.1016/S01406736(20)30736-4](https://doi.org/10.1016/s0140-6736(20)30736-4) |
| 1. Parenting and parent–child home practice during the COVID‑19 pandemic: a case in central China | Sample characteristics | https://doi.org/[10.1038/s41598-023-45726-8](https://doi.org/10.1038/s41598-023-45726-8) |
| 1. Work from home and parenting: Examining the role of work-family conflict and gender during the COVID-19 pandemic | Sample characteristics | https://doi.org/[10.1111/josi.12509](https://doi.org/10.1111/josi.12509) |
| 1. Adjusting Parenting Roles and Work Expectations among Women with Children during COVID-19 | Sample characteristics | https://doi.org/[10.1177/10443894231183609](https://doi.org/10.1177/10443894231183609) |
| 1. A Preliminary Study of COVID-19-related Stressors, Parenting Stress, and Parental Psychological Well-being Among Parents of School-age Children | Sample characteristics | https://doi.org/[10.1007/s10826-022-02321-1](https://doi.org/10.1007/s10826-022-02321-1) |
| 1. Perceptions of Parenting, Parent-Child Activities and Children’s Extracurricular Activities in Times of COVID-19 | Sample characteristics | https://doi.org/[10.1007/s10826-021-02171-3](https://doi.org/10.1007/s10826-021-02171-3) |
| 1. LGBTQ+ College Students' Relationship Satisfaction During the COVID-19 Pandemic | Sample characteristics | https://doi.org/[10.1177/21676968231160305](https://doi.org/10.1177/21676968231160305) |
| 1. Need for Couple's Awareness About Sexual Health in COVID-19 Pandemic | Study characteristics | https://doi.org/[10.5195/cajgh.2020.490](https://doi.org/10.5195/cajgh.2020.490) |
| 1. The role of sexting in couple wellbeing for Italian women during the second wave of the COVID-19 pandemic | Sample characteristics | https://doi.org/[10.3389/fpsyg.2023.1105556](https://doi.org/10.3389/fpsyg.2023.1105556) |
| 1. The Impact of Lockdown on Couples' Sex Lives | Sample characteristics | https://doi.org/10.3390/jcm10071414. |
| 1. "It's splendid once you grow into it:" Client experiences of relational teletherapy in the era of COVID-19 | Target of the Study | https://doi.org/[10.1111/jmft.12508](https://doi.org/10.1111/jmft.12508) |
| 1. Effect of lockdown on mental health in Australia: evidence from a natural experiment analysing a longitudinal probability sample survey | Target of the Study | https://doi.org/[10.1016/S2468-2667(22)00082-2](https://doi.org/10.1016/s2468-2667(22)00082-2) |
| 1. Partners in lockdown: Relationship stress in men and women during the COVID-19 pandemic. | Sample characteristics | https://doi.org/10.1037/cfp0000172 |
| 1. “This Is Not the Hill to Die on. Even if We Literally Could Die on This Hill”: Examining Communication Ecologies of Uncertainty and Family Communication About COVID-19 | Sample characteristics | <https://doi.org/10.1177/0002764221992840> |
| 1. Feeling the Absence of Touch: Distancing, Distress, Regulation, and Relationships in the Context of COVID-19 | Sample characteristics | <https://doi.org/10.1177/026540752110526> |
| 1. Relationship satisfaction in the time of COVID-19: The role of shared reality in perceiving partner support for frontline health-care workers | Sample characteristics | <https://doi.org/10.1177/02654075211020127> |
| 1. Relational turbulence from the COVID-19 pandemic: Within-subjects mediation by romantic partner interdependence | Sample characteristics | <https://doi.org/10.1177/0265407521100013> |
| 1. Examining associations between COVID-19 stressors, intimate partner violence, health, and health behaviors | Sample characteristics | <https://doi.org/10.1177/02654075211012098> |
| 1. Managing Multiple Roles During the COVID-19 Lockdown: Not Men or Women, but Parents as the Emotional “Loser in the Crisis | Sample characteristics | <https://doi.org/10.32872/spb.4347> |
| 1. Arguing about social distancing and family relationships | Sample characteristics | <https://doi.org/10.1177/026540752110407> |
| 1. Day-to-day relational life during the COVID-19 pandemic: Linking mental health, daily relational experiences, and end-of-day outlook | Sample characteristics | <https://doi.org/10.1177/02654075211020> |
| 1. Changes in social relationships during an initial “stay-at-home” phase of the COVID-19 pandemic: A longitudinal survey study in the U.S. | Target of the Study | <https://doi.org/10.1016/j.socscimed.2021.113779> |
| 1. Relational Mobility Predicts Faster Spread of COVID-19: A 39-Country Study | Target of the Study | <https://doi.org/10.1177/095679762095811> |
| 1. Parent–student relational turbulence, support processes, and mental health during the COVID-19 pandemic | Target of the Study  Sample characteristics | <https://doi.org/10.1177/02654075211041658> |
| 1. Relationship difficulties and “technoference” during the COVID-19 pandemic | Sample characteristics | <https://doi.org/10.1177/0265407522109361> |
| 1. Mothers’ Sources and Strategies for Managing COVID-19 Uncertainties during the Early Pandemic Months | Sample characteristics | <https://doi.org/10.1080/15267431.2021.1928135> |
| 1. Spousal interference and relational turbulence during the COVID-19 pandemic | Target of the Study | <https://doi.org/10.1080/08824096.2020.1841621> |
| 1. [Family functioning in an international sample of households reporting adult caregiving during the COVID-19 pandemic.](https://psycnet.apa.org/record/2021-83038-001?doi=1) | Sample characteristics | <https://doi.org/10.1037/fsh0000653> |
| 1. Stress, Dyadic Coping, and Relationship Instability During the COVID-19 Pandemic | Sample characteristics | <https://doi.org/10.1177/02654075211046531> |
| 1. [Clinically significant depression among parents during the COVID-19 pandemic: Examining the protective role of family relationships.](https://psycnet.apa.org/record/2021-55842-001?doi=1) | Target of the Study | <https://doi.org/10.1037/cfp0000175> |
| 1. Relational maintenance, collectivism, and coping strategies among Black populations during COVID-19 | Sample characteristics | <https://doi.org/10.1177/0265407521102509> |
| 1. How families matter for health inequality during the COVID-19 pandemic | Target of the Study | <https://doi.org/10.1111/jftr.12398> |
| 1. Effects of the COVID-19 Pandemic on Parental Burnout and Parenting Practices: Analyses Using a Retrospective Pretest | Sample characteristics | https://doi.org/[10.1177/24705470221114059](https://doi.org/10.1177/24705470221114059) |
| 1. Parental anxiety and form of parenting during the COVID-19 pandemic | Sample characteristics | https://doi.org/[10.1186/s40723-022-00103-2](https://doi.org/10.1186/s40723-022-00103-2) |
| 1. The Role of Fathers' Parenting Stress and His Parenting Styles on Behavior Problems in Children During the Covid-19 Pandemic | Sample characteristics | https://doi.org/[10.2991/ahsr.k.211130.024](https://doi.org/10.2991/ahsr.k.211130.024) |
| 1. [The anxiety level among mothers during the COVID-19 pandemic](https://ejournal.upi.edu/index.php/JOMSIGN/article/view/33491) | Sample characteristics | https://doi.org/10.17509/jomsign.v5i2.33491 |
| 1. Parents and Parenting in the COVID-19 Pandemic: A Review of the COVID-19 Literature. | Study characteristics | https://doi.org/10.1007/978-3-031-15359-4_23 |
| 1. Parent-Child/Adolescent Relationships During the COVID-19 Pandemic | Target of the Study | https://doi.org/10.1007/978-3-031-15359-4_24 |
| 1. Parent satisfaction when schools from home during the COVID-19 pandemic | Sample characteristics | https://doi.org/10.11591/ijphs.v11i3.21223 |
| 1. Conclusion to the special issue: Relationships in the time of COVID-19: Examining the effects of the global pandemic on personal relationships | Study characteristics | <https://doi.org/10.1177/02654075211063536> |
| 1. Now for the Good News: Self-Perceived Positive Effects of the First Pandemic Wave on Romantic Relationships Outweigh the Negative | Sample characteristics | <https://doi.org/10.1177/02654075211050939> |
| 1. Marital distress during COVID-19 pandemic and lockdown: a brief narrative | Study characteristics | https://doi.org/10.25215/0802.257 |
| 1. Relationship Status and Well-Being in the Context of the COVID-19 Pandemic | Sample characteristics | <https://doi.org/10.1177/0192513X221105242> |
| 1. COVID-19 pandemic: influence of relationship status on stress, anxiety, and depression in Canada | Sample | <https://doi.org/10.1017/ipm.2021.1> |
| 1. [Mental health and relationships during the COVID-19 pandemic](https://www.academia.edu/download/89192331/Mental_health_and_relationships.pdf) | Not peer review |  |
| 1. Quarantine, distress and interpersonal relationships during COVID-19 | Sample Characteristics | https://doi.org/[10.1136/gpsych-2020-100385](https://doi.org/10.1136/gpsych-2020-100385) |
| 1. Initial Impacts of COVID-19 on Sex Life and Relationship Quality in Steady Relationships in Britain: Findings from a Large, Quasi-representative Survey (Natsal-COVID) | Sample Characteristics | <https://doi.org/10.1080/00224499.2022.2035663> |
| 1. A Mixed-method Study of Individual, Couple, and Parental Functioning During the State-regulated COVID-19 Lockdown in Spain | Sample Characteristics | <https://doi.org/10.1111/famp.12585> |
| 1. Perceived stress as mediator for longitudinal effects of the COVID-19 lockdown on wellbeing of parents and children | Target of the Study | https://doi.org/10.1038/s41598-021-81720-8 |
| 1. Couples’ Relationships in the Age of COVID-19 | Target of the Study Sample Characteristics Study Characteristics | https://www. wccbt. org/Downloads/WCCBT_e-News_Sept-2020. pdf. |
| 1. Supporting Romantic Relationships During COVID-19 Using Virtual Couple Therapy | Target of the Study | <https://doi.org/10.1016/j.cbpra.2021.02.002> |
| 1. Rituals in the Time of COVID-19: Imagination, Responsiveness, and the Human Spirit | Target of the Study | h[ttps://doi.org/10.1111/famp.12581](https://doi.org/10.1111/famp.12581) |
| 1. Couples Therapists’ Attitudes Toward Online Therapy During the COVID-19 Crisis | Target of the Study | <https://doi.org/10.1111/famp.12647> |
| 1. Couple teletherapy in the era of COVID-19: Experiences and recommendations | Target of the Study | <https://doi.org/10.1111/jmft.12501> |
| 1. Helping Couples Connect during the COVID-19 Pandemic: A Pilot Randomised Controlled Trial of an Awareness, Courage, and Love Intervention | Target of the Study  Sample Characteristics | <https://doi.org/10.1111/aphw.12241> |
| 1. Couple Connectedness in the Time of COVID-19 | Sample Characteristics Study Characteristics | <https://doi.org/10.1177/10664807221082397> |
| 1. “I have turned into a foreman here at home”: Families and work–life balance in times of COVID-19 in a gender equality paradise | Sample Characteristics | <https://doi.org/10.1111/gwao.12552> |
| 1. [Healthy Relationships Utah during COVID-19](https://digitalcommons.usu.edu/oiq/vol1/iss1/2/) | Sample Characteristics | https://doi.org/https://doi.org/10.26077/8564-dda6 |
| 1. Furloughs, Teleworking and Other Work Situations during the COVID-19 Lockdown: Impact on Mental Well-Being | Sample Characteristics | https://doi.org/10.3390/ijerph18062898 |
| 1. COVID-19 and the rise of intimate partner violence | Sample Characteristics | https://doi.org/10.1016/j.worlddev.2020.105217 |
| 1. Intimate partner violence during the COVID-19 pandemic in Western and Southern European countries | Sample Characteristics | https://doi:10.1093/eurpub/ckab093 |
| 1. Patterns, prevalence and risk factors of intimate partner violence and its association with mental health status during COVID-19: a cross-sectional study on early married female adolescents in Khulna district, Bangladesh | Sample Characteristics | https://doi:10.1136/bmjopen-2023-072279 |
| 1. Telework and Work–Family Conflict during COVID-19 Lockdown in Portugal: The Influence of Job-Related Factors | Sample Characteristics | https://doi.org/10.3390/admsci11030103 |
| 1. Work–family and family–work conflict and stress in times of COVID-19 | Sample Characteristics | https://doi.10.3389/fpsyg.2022.951149 |
| 1. The Influence of Work–Family Conflict on Burnout during the COVID-19 Pandemic: The Effect of Teleworking Overload | Sample Characteristics | https://doi.org/10.3390/ijerph181910302 |
| 1. Dual-earner Parent Couples’ Work and Care during COVID-19 | Sample Characteristics | https://doi.org/10.1111/gwao.12497 |
| 1. Dual-earner couples during the pandemic: Spillover and   crossover | Study characteristics | https://doi.org/10.1017/iop.2021.56 |
| 1. The Effects of Depression and Fear in   Dual-Income Parents onWork-Family  Conflict During the COVID-19 Pandemic | Study characteristics | https://doi.org/10.1177/21582440231157662 |
| 1. COVID-19 pandemic and the quality of couples’ sexual relationships | Study characteristics | https://doi.org/10.38053/acmj.848051 |
| 1. The COVID-19 Pandemic: A Family Affair | Study characteristics | <https://doi.org/10.1177/1074840720920883> |
| 1. Family Resilience during COVID-19 Pandemic: A Literature Review | Study characteristics | <https://doi.org/10.1177/10664807211023875> |
| 1. Family Caregiving During the COVID-19 Pandemic | Study characteristics | https://doi.org/10.1093/geront/gnab049 |
| 1. The Impact of COVID-19 Pandemic on Family Well-Being: A Literature Review | Study characteristics | <https://doi.org/10.1177/10664807221131006> |
| 1. The invisible workforce during the COVID-19 pandemic: Family carers at the frontline | Study characteristics | https://doi.org/[10.12688/hrbopenres.13059.1](https://doi.org/10.12688/hrbopenres.13059.1) |
| 1. The Influence of the Ongoing COVID-19 Pandemic on Family Violence in China | Study characteristics | https://doi.org/10.1007/s10896-020-00196-8 |
| 1. COVID-19 and family violence: Is this a perfect storm? | Study characteristics | <https://doi.org/10.1111/inm.12876> |
| 1. Family functioning and mental wellbeing impairment during initial quarantining for the COVID-19 pandemic: A study of Canadian families | Sample characteristics | https://doi.org/10.1007/s12144-021-02689-1 |
| 1. Family Functioning in the Time of COVID-19 Among Economically Vulnerable Families: Risks and Protective Factors | Sample characteristics | <https://doi.org/10.3389/fpsyg.2021.730447> |
| 1. Family functioning and mental health among secondary vocational students during the COVID-19 epidemic: A moderated mediation model | Sample characteristics | <https://doi.org/10.1016/j.paid.2020.110490> |
| 1. Impact of family functioning on mental health problems of college students in China during COVID-19 pandemic and moderating role of coping style: a longitudinal study. | Sample characteristics | https://doi.org/10.1186/s12888-023-04717-9 |
| 1. Family Function and Child Adjustment Difficulties in the COVID-19 Pandemic: An International Study | Sample characteristics | https://doi.org [10.3390/ijerph182111136](https://www.mdpi.com/1660-4601/18/21/11136) |
| 1. Effects of Coronavirus Disease 2019 (COVID-19) on Family Functioning | Study characteristics | https://doi.org[10.1016/j.jpeds.2021.06.082](https://doi.org/10.1016/j.jpeds.2021.06.082) |
| 1. The Social and Economic Impact of Covid-19 on Family Functioning and Well-Being: Where do we go from here? | Study characteristics | https://doi.org/10.1007/s10834-022-09848-x |
| 1. Impacts of COVID-19 and partial lockdown on family functioning, intergenerational communication and associated psychosocial factors among young adults in Singapore | Sample characteristics | https://doi.org/10.1186/s12888-021-03599-z |
| 1. Family functioning buffers the consequences of the COVID-19 pandemic for children’s quality of life and loneliness | Sample characteristics | <https://doi.org/10.3389/fpsyg.2022.1079848> |
| 1. Investigating the Impact of Isolation During COVID-19 on Family Functioning – An Australian Snapshot | Sample characteristics | <https://doi.org/10.3389/fpsyg.2021.722161> |
| 1. Family Functioning and Psychological Well-Being: The Mediating Role of Coping Strategies during Covid-19 Lockdown in Pakistan | Sample characteristics | https://doi.org10.24425/ppb.2021.137259 |
| 1. Exploring the interplay between individual and family functioning during the COVID-19 pandemic: a cross-sectional study | Sample characteristics | https://doi.org/10.1007/s12144-024-06058-6 |
| 1. Correlation of Sexual Behavior Change, Family Function, and Male-Female Intimacy Among Adults Aged 18-44 Years During COVID-19 Epidemic | Sample characteristics | <https://doi.org/10.1016/j.esxm.2020.100301> |
| 1. The Role Of Family Functioning In The Quarter-Life Crisis In Early Adulthood During The Covid-19 Pandemic | Sample characteristics | p-ISSN: 2460-8750 e-ISSN: 2615-1731 https://doi.org/10.26858/talenta.v7i1.27184 |
| 1. The Role Of Family Functioning In The Quarter-Life Crisis In Early Adulthood During The Covid-19 Pandemic | Sample characteristics | https://doi.org/10.26858/talenta.v7i1.27184 |
| 1. Effects of Covid-19 lockdown on parental functioning in vulnerable families | Sample characteristics | https://doi.org/10.1111/jomf.12789 |
| 1. Child, parent, and family mental health and functioning in Australia during COVID‑19: comparison to pre‑pandemic data | Sample characteristics | https://doi.org/10.1007/s00787-021-01861-z |
| 1. Family functions, social support and quality of life among elderly during pandemic COVID-19: A cross-sectional study | Sample characteristics | https://doi.org/10.53730/ijhs.v6nS4.6281 |
| 1. [COVID-19 pandemic lockdown responses from an emotional perspective: Family function as a differential pattern among older adults.](https://www.behavioralpsycho.com/wp-content/uploads/2021/09/07.Lopez_29-2En.pdf) | Sample characteristics | https://doi.org/10.51668/bp.8321207n |
| 1. “We Are Staying at Home.” Association of Self-perceptions of Aging, Personal and Family Resources, and Loneliness with Psychological Distress During the Lock-Down Period of COVID-19 | Sample characteristics | https://doi.org/10.1093/geronb/gbaa048 |
| 1. The Negative Impacts of COVID-19 Containment Measures on South African Families - Overview and Recommendations | Target of the Study | https://doi.org/[10.2174/1874944502114010233](http://dx.doi.org/10.2174/1874944502114010233) |
| 1. The impact of psychological flexibility on family dynamics amidst the COVID-19 pandemic: A longitudinal perspective | Sample characteristics | <https://doi.org/10.1016/j.jcbs.2022.08.011> |
| 1. Psychological well-being among older adults during the COVID-19 outbreak: a comparative study of the young–old and the old–old adults | Sample characteristics | https://doi.org/10.1017/S1041610220000964 |
| 1. Longitudinal Impact of the COVID-19 Pandemic on Older Adults' Wellbeing | Sample characteristics | <https://doi.org/10.3389/fpsyt.2022.837533> |
| 1. The impact of the COVID‑19 pandemic on wellbeing and cognitive functioning of older adults | Sample characteristics | \| https://doi.org/10.1038/s41598-021-84127-7 |
| 1. Decline in Marriage Associated with the COVID-19 Pandemic in the United States | Target of the Study | [https://doi.org/10.1177/23780231209803](https://doi.org/10.1177/2378023120980328) |
| 1. Settling down without settling: Perceived changes in partner preferences in response to COVID-19 | Sample characteristics | <https://doi.org/10.1177/02654075211011527> |
| 1. Changes in Sex Life among People in Taiwan During the COVID-19 Pandemic: The Roles of Risk Perception, General Anxiety, and Demographic Characteristics | Sample characteristics | https://doi.10.3390/ijerph17165822 |
| 1. The work–family balance of British working women during the COVID-19 pandemic | Sample characteristics | https://doi.10.1108/JWAM-07-2020-0036 |
| 1. Family and Family Relations at the Time of COVID-19: An Introduction | Study characteristics | https://doi.10.13136/isr.v10i3S.393 |
| 1. The Impact of COVID-19 on Family Relationships in Italy: Withdrawal on the Nuclear Family | Sample characteristics | https://doi.10.13136/isr.v10i3S.394 |
| 1. Brief Research Report: The Association Between Educational Experiences and Covid-19 Pandemic-Related Variables, and Mental Health Among Children and Adolescents | Target of the Study | <https://doi.org/10.3389/fpsyt.2021.647456> |
| 1. “Did You Bring It Home with You?” A Qualitative Investigation of the Impacts of the COVID-19 Pandemic on Victorian Frontline Healthcare Workers and Their Families | Sample characteristics | <https://doi.org/10.3390/ijerph19084897> |
| 1. Mental health issues among health care workers during the COVID-19 pandemic – A study from India | Sample characteristics | <https://doi.org/10.1016/j.ajp.2021.102626> |
| 1. Life Interrupted: Family Routines Buffer Stress during the COVID-19 Pandemic | Sample characteristics | https://doi.org/10.1007/s10826-021-02063-6 |
| 1. Impact of COVID-19 pandemic on mental health: An international study | Sample characteristics | <https://doi.org/10.1371/journal.pone.0244809> |
| 1. Family and Mental Health During the Confinement Due to the COVID-19 Pandemic in Spain: The Perspective of the Counselors Participating in Psychological Helpline Services | Target of the Study | <https://doi.org/10.3138/jcfs.51.3-4.014> |
| 1. Parents’ perceived stress and children’s adjustment during the COVID-19 lockdown in Italy: The mediating role of family resilience | Sample characteristics | <https://doi.org/10.1111/fare.12716> |
| 1. A Qualitative Investigation of the Impact of COVID-19 on United States’ Frontline Health Care Workers and the Perceived Impact on Their Family Members | Sample characteristics | <https://doi.org/10.3390/ijerph191710483> |
| 1. The Effect of The Belief System, Family Organizations and Family Communication on Covid-19 Prevention Behavior: The Perspective of Family Resilience | Sample characteristics | https://doi.org/10.24815/ijdm.v4i2.20411 |
| 1. Perceived stress, resources and adaptation in relation to the COVID-19 lockdown in Spanish foster and non-foster families | Sample characteristics | <https://doi.org/10.1111/cfs.12871> |
| 1. Relationship satisfaction in the time of COVID-19: The role of shared reality in perceiving partner support for frontline health-care workers | Sample characteristics | <https://doi.org/10.1177/02654075211020127> |
| 1. Relationship and Sexual Quality in the Wake of COVID-19: Effects of Individual Regulatory Focus and Shared Concerns over the Pandemic | Sample characteristics | <https://doi.org/10.3390/ejihpe13020035> |
| 1. Trajectories of relationship and sexual satisfaction over2 years in the Covid-19 pandemic: A latent class analysis | Sample characteristics | <https://10.1111/jopy.12928> |
| 1. The Impact of Loosening COVID-19 Restrictions and Live-in Partner Status on Sexual and Mental Health in a Canadian Sample | Sample characteristics | <https://doi.org/10.1080/19317611.2022.2163446> |
| 1. Psychophysical Impact of COVID-19 Pandemic and Same-Sex Couples’ Conflict: The Mediating Effect of Internalized Sexual Stigma | Sample characteristics | <https://doi.org/10.3389/fpsyg.2022.860260> |
| 1. Changes in Solo and Partnered Sexual Behaviors during the COVID-19 Pandemic: Findings from a U.S. Probability Survey | Sample characteristics | https://doi.org/10.1101/2020.06.09.20125609 |
| 1. Perceptions of Relationship Quality Before and During COVID-19 Pandemic Among Young Sexual Minority Men in Romantic Relationships | Sample characteristics | https://doi.org/10.1007/s10508-021-02254-8 |
| 1. Satisfaction with life and psychological distress during the COVID-19 pandemic: An Egyptian online cross-sectional study | Sample characteristics | https://doi.org/[10.4102/phcfm.v14i1.2896](https://doi.org/10.4102/phcfm.v14i1.2896) |
| 1. A Prospective Study of Mental Health, Well-Being, and Substance Use During the Initial COVID-19 Pandemic Surge | Sample characteristics | https://doi.org/[10.1177/21677026211013499](https://doi.org/10.1177/21677026211013499) |
| 1. The Impact of the COVID-19 Pandemic on Sexual Behaviors: Findings From a National Survey in the United States | Sample characteristics | https://doi.org/[10.1016/j.jsxm.2021.08.008](https://doi.org/10.1016/j.jsxm.2021.08.008) |
| 1. Love in the Time of COVID-19: A Multi-Wave Study Examining the Salience of Sexual and Relationship Health During the COVID-19 Pandemic | Sample characteristics | https://doi.org/[10.1007/s10508-021-02208-0](https://doi.org/10.1007/s10508-021-02208-0) |
| 1. A profile analysis of COVID-19 stress-related reactions: The importance of early childhood abuse, psychopathology, and interpersonal relationships | Sample characteristics | https://doi.org/[10.1016/j.chiabu.2021.105442](https://doi.org/10.1016/j.chiabu.2021.105442) |
| 1. COVID-19 Infection among Family and Friends: The Psychological Impact on Non-Infected Persons | Sample characteristics | https://doi.org/[10.3390/brainsci12091123](https://doi.org/10.3390/brainsci12091123) |
| 1. COVID-19 and Family Distancing Efforts: Contextual Demographic and Family Conflict Correlates | Sample characteristics | https://doi.org/[10.1177/0192513X211055123](https://doi.org/10.1177/0192513x211055123) |
| 1. Family Satisfaction With Critical Care: Before and After the COVID-19 Outbreak | Sample characteristics | https://doi.org/[10.7759/cureus.33853](https://doi.org/10.7759/cureus.33853) |
| 1. Depression, Anxiety, Perceived Stress and Family Support in COVID-19 Patients | Sample characteristics | https://doi.org/[10.18502/ijps.v17i3.9725](https://doi.org/10.18502/ijps.v17i3.9725) |
| 1. COVID-19 and Work-Family Conflicts in Germany: Risks and Chances Across Gender and Parenthood | Sample characteristics | https://doi.org/[10.3389/fsoc.2021.780740](https://doi.org/10.3389/fsoc.2021.780740) |
| 1. Gender, work-family conflict and depressive symptoms during the COVID-19 pandemic among Quebec graduate students | Sample characteristics | https://doi.org/[10.1016/j.pmedr.2021.101568](https://doi.org/10.1016/j.pmedr.2021.101568) |
| 1. Family Resilience and Psychological Responses to COVID-19: A Study of Concordance and Dyadic Effects in Singapore Households | Sample characteristics | https://doi.org/[10.3389/fpsyg.2022.770927](https://doi.org/10.3389/fpsyg.2022.770927) |
| 1. Family Cohesion and Stress Consequences Among Chinese College Students During COVID-19 Pandemic: A Moderated Mediation Model | Target of the Study | https://doi.org/[10.3389/fpubh.2021.703899](https://doi.org/10.3389/fpubh.2021.703899) |
| 1. Alcohol use, sleep, and depression among family caregivers in the time of COVID-19 | Sample characteristics | https://doi.org/[10.1016/j.alcohol.2022.04.002](https://doi.org/10.1016/j.alcohol.2022.04.002) |
| 1. COVID-19 and Mental Health of Young Adult Children in China: Economic Impact, Family Dynamics, and Resilience | Sample characteristics  Target of the Study | https://doi.org/[10.1111/fare.12573](https://doi.org/10.1111/fare.12573) |
| 1. Internalizing symptoms and family functioning predict adolescent depressive symptoms during COVID-19: A longitudinal study in a community sample | Target of the Study | https://doi.org/[10.1371/journal.pone.0264962](https://doi.org/10.1371/journal.pone.0264962) |
| 1. The Relationships Between Family Characteristics and Undergraduate Students' COVID-19 Responses: A Cross-Sectional Study in China | Target of the Study | <https://doi.org>[10.3389/fpubh.2022.873696](https://doi.org/10.3389/fpubh.2022.873696) |
| 1. The effects of COVID-19 stressors and family life on anxiety and depression one-year into the COVID-19 pandemic | Sample characteristics | https://doi.org[10.1111/famp.12771](https://doi.org/10.1111/famp.12771) |
| 1. Is the mental health of young students and their family members affected during the quarantine period? Evidence from the COVID-19 pandemic in Albania | Sample characteristics | https://doi.org/[10.1111/jpm.12672](https://doi.org/10.1111/jpm.12672) |
| 1. Impact of COVID-19 on family planning | Study characteristics | https://doi.org/[10.18332/ejm/137484](https://doi.org/10.18332/ejm/137484) |
| 1. Psychological state, family functioning and coping strategies among undergraduate students in a Nigerian University during the COVID-19 lockdown | Sample characteristics | https://doi.org/[10.15167/2421-4248/jpmh2021.62.2.1798](https://doi.org/10.15167/2421-4248/jpmh2021.62.2.1798) |
| 1. The role of family communication patterns in intergenerational COVID-19 discussions and preventive behaviors: a social cognitive approach | Sample characteristics | https://doi.org/[10.1186/s40359-023-01331-y](https://doi.org/10.1186/s40359-023-01331-y) |
| 1. Black American Fathers Employed in Higher-Risk Contexts for Contracting COVID-19: Implications for Individual Wellbeing and Work-Family Spillover | Sample characteristics | https://doi.org/[10.1177/15579883211005617](https://doi.org/10.1177/15579883211005617) |
| 1. Family Resilience and Dyadic Coping during the Outbreak of the COVID-19 Pandemic in Italy: Their Protective Role in Hedonic and Eudaimonic Well-Being | Sample characteristics | https://doi.org/[10.3390/ijerph20186719](https://doi.org/10.3390/ijerph20186719) |
| 1. Family Functioning and Optimism as Protective Factors of Life Satisfaction Among Stroke Patients During the COVID-19 Epidemic in Shenyang, China | Sample characteristics | https://doi.org/[10.3389/fpubh.2022.738634](https://doi.org/10.3389/fpubh.2022.738634) |
| 1. The Psychological Consequences of COVID-19 Fear and the Moderator Effects of Individuals' Underlying Illness and Witnessing Infected Friends and Family | Sample characteristics | https://doi.org/[10.3390/ijerph18041836](https://doi.org/10.3390/ijerph18041836) |
| 1. War on Two Fronts: Experience of Children with Cancer and Their Family During COVID-19 Pandemic in Iran | Sample characteristics | https://doi.org/[10.1016/j.pedn.2020.10.024](https://doi.org/10.1016/j.pedn.2020.10.024) |
| 1. The relationship between nurses' work stress levels and work-family conflict during the COVID-19 pandemic and the affecting factors: A study from Turkey | Sample characteristics | https://doi.org/[10.1016/j.apnu.2022.12.015](https://doi.org/10.1016/j.apnu.2022.12.015) |
| 1. Effects of Socioeconomic Status, Parental Stress, and Family Support on Children's Physical and Emotional Health During the COVID-19 Pandemic | Target of the Study | https://doi.org/[10.1007/s10826-022-02339-5](https://doi.org/10.1007/s10826-022-02339-5) |
| 1. A Qualitative Investigation of the Impact of COVID-19 on United States' Frontline Health Care Workers and the Perceived Impact on Their Family Members | Sample characteristics | https://doi.org/[10.3390/ijerph191710483](https://doi.org/10.3390/ijerph191710483) |
| 1. Parenting and pandemic pressures: Examining nuances in parent, child, and family well-being concerns during COVID-19 in a Canadian sample | Sample characteristics | https://doi.org/[10.3389/fepid.2023.1073811](https://doi.org/10.3389/fepid.2023.1073811) |
| 1. Investigating the burden of disease dimensions (time-dependent, developmental, physical, social and emotional) among family caregivers with COVID-19 patients in Iran | Sample characteristics | https://doi.org/[10.1186/s12875-022-01772-1](https://doi.org/10.1186/s12875-022-01772-1) |
| 1. Evaluating the Efficacy of the Family Check-Up Online to Improve Parent Mental Health and Family Functioning in Response to the COVID-19 Pandemic: A Randomized Clinical Trial | Sample characteristics | https://doi.org/[10.1007/s10935-023-00727-1](https://doi.org/10.1007/s10935-023-00727-1) |
| 1. Psychological distress, employment, and family functioning during the COVID-19 outbreak among recent immigrant families in Israel: Moderating roles of COVID-19 prevalence | Sample characteristics | https://doi.org/[10.1371/journal.pone.0277757](https://doi.org/10.1371/journal.pone.0277757) |
| 1. Resilience and mental health during the COVID-19 pandemic: Findings from Minnesota and Hong Kong | Sample characteristics | <https://doi.org/10.1016/j.jad.2021.08.144> |
| 1. Parents' Perceived Impact of the Societal Lockdown of COVID-19 on Family Well-Being and on the Emotional and Behavioral State of Walloon Belgian Children Aged 4 to 13 Years: An Exploratory Study | Target of the Study | https://doi.org/[10.5334/pb.1059](https://doi.org/10.5334/pb.1059) |
| 1. Perceived Changes in Family Life During COVID-19: The Role of Family Size | Sample characteristics | https://doi.org/[10.1111/fare.12579](https://doi.org/10.1111/fare.12579) |
| 1. The effect of COVID-19 epidemic on the mental health of nurses' family members | Sample characteristics | https://doi.org/10.4103/jehp.jehp_1399_20 |
| 1. A U.S. National Study of Family Resilience During the COVID-19 Pandemic | Sample characteristics | https://doi.org/[10.1007/s10826-023-02581-5](https://doi.org/10.1007/s10826-023-02581-5) |
| 1. Mental Health before and during the COVID-19 Pandemic: The Role of Partnership and Parenthood Status in Growing Disparities between Types of Families | Sample characteristics | https://doi.org/[10.1177/00221465221109195](https://doi.org/10.1177/00221465221109195) |
| 1. The Influence of Work-Family Conflict on Burnout during the COVID-19 Pandemic: The Effect of Teleworking Overload | Sample characteristics | https://doi.org/[10.3390/ijerph181910302](https://doi.org/10.3390/ijerph181910302) |
